# Therapeutic antidepressant potential of a conjugated siRNA silencing the serotonin transporter after intranasal administration

**DOI:** 10.1038/mp.2015.80

**Published:** 2015-06-23

**Authors:** A Ferrés-Coy, M Galofré, F Pilar-Cuéllar, R Vidal, V Paz, E Ruiz-Bronchal, L Campa, Á Pazos, J R Caso, J C Leza, G Alvarado, A Montefeltro, E M Valdizán, F Artigas, A Bortolozzi

**Affiliations:** 1 Institut d'Investigacions Biomèdiques August Pi i Sunyer (IDIBAPS), Barcelona, Spain; 2 Department of Neurochemistry and Neuropharmacology, IIBB-CSIC (Consejo Superior de Investigaciones Científicas), Barcelona, Spain; 3 Centro de Investigación Biomédica en Red de Salud Mental (CIBERSAM), ISCIII, Madrid, Spain; 4 Institute of Biomedicine and Biotechnology of Cantabria (IBBTEC; UC-CISC-SODERCAN), Santander, Spain; 5 Department of Pharmacology, School of Medicine, Universidad Complutense and IIS Hospital 12 de Octubre, Madrid, Spain; 6 n-Life Therapeutics, S.L., Granada, Spain

## Abstract

Major depression brings about a heavy socio-economic burden worldwide due to its high prevalence and the low efficacy of antidepressant drugs, mostly inhibiting the serotonin transporter (SERT). As a result, ~80% of patients show recurrent or chronic depression, resulting in a poor quality of life and increased suicide risk. RNA interference (RNAi) strategies have been preliminarily used to evoke antidepressant-like responses in experimental animals. However, the main limitation for the medical use of RNAi is the extreme difficulty to deliver oligonucleotides to selected neurons/systems in the mammalian brain. Here we show that the intranasal administration of a sertraline-conjugated small interfering RNA (C-SERT-siRNA) silenced SERT expression/function and evoked fast antidepressant-like responses in mice. After crossing the permeable olfactory epithelium, the sertraline-conjugated-siRNA was internalized and transported to serotonin cell bodies by deep Rab-7-associated endomembrane vesicles. Seven-day C-SERT-siRNA evoked similar or more marked responses than 28-day fluoxetine treatment. Hence, C-SERT-siRNA (i) downregulated 5-HT_1A_-autoreceptors and facilitated forebrain serotonin neurotransmission, (ii) accelerated the proliferation of neuronal precursors and (iii) increased hippocampal complexity and plasticity. Further, short-term C-SERT-siRNA reversed depressive-like behaviors in corticosterone-treated mice. The present results show the feasibility of evoking antidepressant-like responses by selectively targeting neuronal populations with appropriate siRNA strategies, opening a way for further translational studies.

## Introduction

Major depressive disorder (MDD) is a severe, chronic and life-threatening disease with a high incidence; affecting ca. 120 million people worldwide.^1, 2, 3^ The midbrain serotonin (5-hydroxytryptamine (5-HT)) system has a critical role in many brain functions, including mood control. Derangements of serotonin pathway are involved in MDD, and most antidepressant drugs aim to increase serotonergic function.^4^ Serotonin transporter (SERT) is a key player in MDD, by controlling the active 5-HT fraction and, being the target of most prescribed antidepressant drugs, the selective serotonin reuptake inhibitors (SSRI) and the selective serotonin and norepinephrine reuptake inhibitors (SNRI).^5, 6^ These drugs need to be administered for long time before clinical improvement emerges, and they fully remit depressive symptoms in only one-third of patients leaving a large proportion of people with partial or incomplete clinical responses.^7, 8^ For these reasons, there is an urgent need to improve antidepressant treatments.

Chronic—but not acute—SSRI treatments elicit a series of neurobiological changes relevant for antidepressant activity. Hence, chronic SSRI treatments downregulates SERT, increasing forebrain serotonergic neurotransmission and neuronal plasticity in the hippocampus,^9, 10, 11, 12^ although the precise mechanisms involved remain uncertain. Likewise, chronic SSRI treatments internalize SERT and reduce SERT-binding sites without affecting SERT mRNA levels.^9, 10, 13, 14^ In particular, fluoxetine (FLX) promotes the biogenesis of microRNA-16, resulting in a downstream repression of SERT levels in mouse 5-HT neurons, accompanied by antidepressant-like effects in the chronic mild stress and forced-swim animal models.^15^

Altogether, these data uncover the functional significance of SERT downregulation in mediating antidepressant responses. The identification of intracellular networks underlying SERT downregulation may be a new target for the development of fast-acting antidepressants. Hence, exogenous small interfering RNAs (siRNAs) have been preliminarily investigated as potential therapeutic tools to silence the expression of critical genes in 5-HT neurons.^16, 17, 18^ Intracerebral treatments with siRNA against SERT—or their related antisense oligonucleotides—significantly decreased SERT expression and function in the rodent brain and evoked cellular and behavioral responses predictive of clinical antidepressant activity.^16, 17, 19^ Despite these exciting prospects, the utility of RNA interference (RNAi)-based silencing strategies for MDD treatment is severely compromised by the extreme difficulty to deliver oligonucleotide sequences to their neuronal functional sites, due to the need to cross several biological barriers after administration and the evident complexity of the mammalian brain.^20, 21^

Here we have used targeted delivery of a sertraline ligand-conjugated siRNA directed against SERT (C-SERT-siRNA) to downregulate SERT expression selectively in raphe 5-HT neurons. We show that C-SERT-siRNA silenced SERT expression/function and evoked fast and robust antidepressant-like responses after intranasal (i.n.) administration in mice. Moreover, it reversed the depressive-like behavior in corticosterone-treated mice due to the increased 5-HT signaling and synaptic plasticity. These results highlight the potential of RNAi-based antidepressant therapies targeting genes linked to antidepressant responses, such as SERT or the 5-HT_1A_-autoreceptor^18^ through a clinically feasible (i.n.) administration route.

## Materials and methods

### Animals

Male C57BL/6J mice (10–14 weeks; Charles River, Lyon, France) were housed under controlled conditions (22±1 °C; 12-h light/dark cycle) with food and water available *ad libitum*. Animal procedures were conducted in accordance with standard ethical guidelines (EU regulations L35/118/12/1986) and approved by the local ethical committee.

### Conjugated siRNA synthesis

The synthesis and purification of sertraline-conjugated siRNA directed against SERT (C-SERT-siRNA, nt: 1230–1250, GenBank accession NM_010484) and sertraline-conjugated nonsense siRNA (C-NS-siRNA) molecules were performed by nLife Therapeutics S.L. (Granada, Spain).^18^ Details are shown in Supplementary Information.

To study *in vivo* intracellular distribution and incorporation of conjugated siRNA into 5-HT neurons, C-NS-siRNA was additionally labeled with alexa488 in the antisense strand (A488-C-NS-siRNA). We used C-NS-siRNA instead of C-SERT-siRNA to examine the brain distribution after i.n. administration because C-SERT-siRNA reduces SERT expression (see Results section), this compromising the penetration of new doses into 5-HT neurons through SERT. Along these lines, we assumed that the main factor conferring the neuronal target selectivity was the presence of covalently bound sertraline rather than the oligonucleotide sequence. Stock solutions of all siRNAs were prepared in RNAse-free water and stored at −20 °C until use. Sequences are shown in Supplementary Table S1.

### Treatments

For i.n. administration, mice were slightly anesthetized by 2% isoflurane inhalation and placed in a supine position.^18^ A 5-μl drop of phosphate-buffered saline (PBS) or conjugated siRNA (C-NS-siRNA and C-SERT-siRNA) was applied alternatively to each nostril once daily. A total of 10 μl of solution containing 30 μg (2.1 nmol day^−1^) of conjugated siRNA was delivered for 1, 4 or 7 days, and mice were killed at 1, 3, 7 or 15 days after last administration. To evaluate the C-SERT-siRNA efficacy on SERT knockdown, mice were i.n. treated with the C-SERT-siRNA at 10, 30 or 100 μg day^−1^ (0.7, 2.1 or 7 nmol day^−1^, respectively) during 7 days and were killed 24 h after last administration.

FLX (Tocris, Madrid, Spain) was administered once daily at 10 mg kg^−1^, intraperitoneally (i.p.), for 7 or 28 days. Mice were killed at 24 h after last administration. Control mice received saline.

Corticosterone (Cortico, Sigma-Aldrich, Madrid, Spain) was dissolved in commercial mineral water and brought to a pH 7.0–7.4 with HCl. Group-housed mice were presented with Cortico solution for 28 or 49 days at: 30 μg ml^−1^ during 15 days (resulting in a dose of approximately 6.6 mg kg^−1^ day^−1^, p.o.), followed by 15 μg ml^−1^ (2.7 mg kg^−1^ day^−1^) during 3 days and 7.5 μg ml^−1^ (1.1 mg kg^−1^ day^−1^) during 10 or 31 days to allow a gradual recovery of endogenous corticosterone plasma level.^22, 23^ Cortico solutions were no more than 3 days old and mantained in opaque bottles to protect it from light. From day 21, one group of animals treated with Cortico received daily i.n. PBS, C-NS-siRNA or C-SERT-siRNA for 7 days. Another group of mice treated with Cortico received i.p. injections of saline or FLX for 7 or 28 days (Supplementary Figure S1).

### Plasma corticosterone levels

Posttreatment corticosterone was measured in plasma obtained from blood samples after cardiac puncture at 1500–1600 hours using trisodium citrate as anticoagulant. After blood centrifugation at 1000 *g* for 15 min, all plasma samples were stored at −80 °C before assay by using a commercially available kit by radioimmunoassay of ^125^I-labeled rat corticosterone (Coat-A-Count, Siemens, Healthcare Diagnostics, Berkeley, CA, USA). A gamma counter (Perkin Elmer Wallac Wizard 1470, Turku, Finland) was used to measure radioactivity of the samples.^24^

### *In situ* hybridization

Mice were killed by pentobarbital overdose, and the brains were rapidly removed, frozen on dry ice and stored at −80 ºC. Coronal tissue sections (14-μm thick) were cut using a microtome-cryostat (HM500-OM, Microm, Walldorf, Germany), thaw-mounted onto 3-aminopropyltriethoxysilane (Sigma-Aldrich)-coated slides and kept at −20 °C until use. Antisense oligoprobes were complementary to bases: SERT/820-863 (GenBank accession NM_010484.1), serotonin1A receptor-5-HT_1A_R/1780-1827 (NM_008308), tryptophan hydroxylase-2 (TPH_2_)/360-410 (NM_173391), brain-derived neurotrophic factor (BDNF)/1188-1238 (NM_007540), vascular endothelial growth factor (VEGF)/2217-2267 (NM_001025250), activity-regulated cytoskeletal protein (ARC)/1990-2040 (NM_018790), TRKB receptor/1075-1124 (NM_001025074), PSD-95/76-120 (D50621), and neuritin/408-448 (NM_153529), respectively (Göttingen, Germany). Oligonucleotides were individually labeled (2 pmol) at the 3'-end with [^33^P]-dATP (>2500 Ci mmol^−1^; DuPont-NEN, Boston, MA, USA) using terminal deoxynucleotidyltransferase (TdT, Calbiochem, La Jolla, CA, USA). Sections were hybridized as previously described.^17, 18, 25^ Details are shown in Supplementary Information.

### Autoradiographic studies

The autoradiographic binding assays for 5-HT_1A_R, SERT and norepinephrine transporter were performed using the following radioligands: (a) [^3^H]-8-OH-DPAT (233 Ci mmol^−1^), (b) [^3^H]-citalopram (70 Ci mmol^−1^) and (c) [^3^H]-nisoxetine (85 Ci mmol^−1^), respectively (Perkin-Elmer, Madrid, Spain) as described previously.^17^ The experimental conditions are summarized in Supplementary Table S2. For 5-HT_1A_R-stimulated [^35^S]GTPγS autoradiography, coronal dorsal raphe nucleus (DR) sections were labeled with 0.04 nM [^35^S]GTPγS.^17^ Details are shown in Supplementary Information.

### Quantitative image analysis of film autoradiograms

Autoradiograms were analyzed and relative optical densities (ROD) were obtained using a computer-assisted image analyzer (MCID, Mering, Germany). The system was calibrated with ^3^H- or ^14^C-microscales standards to obtain fmol mg^−1^ protein equivalents from ROD data. The slide background and non-specific densities were subtracted. ROD were evaluated in two or three adjacent sections by duplicate of each mouse and averaged to obtain individual values. MCID system was also used to acquire pseudocolor images. Black and white photographs were taken from autoradiograms using a Wild 420 microscope (Leica, Heerbrugg, Germany) equipped with Nikon DXM1200F digital camera and ACT-1 Nikon software (Soft Imaging System Gmbh, Münster, Germany). Images were processed with Photoshop (Adobe Systems, Mountain View, CA, USA) by using identical values for contrast and brightness.

### 5-Bromo-2'-deoxyuridine (BrdU) administration

BrdU was purchased from Sigma-Aldrich and dissolved in saline solution. The last day of antidepressant treatment, mice were injected with 4 × 75 mg kg^−1^ BrdU, i.p., every 2h and were killed 24h later in according to Santarelli *et al.*^11^

### Immunohistochemistry

Mice were anesthetized with pentobarbital and transcardially perfused with 4% paraformaldehyde in sodium–phosphate buffer (pH 7.4). Brains were collected, postfixed 24 h at 4 °C in the same solution and then placed in gradient sucrose 10–30% for 3 days at 4 °C. After cryopreservation, serial 30-μm thick sections were cut through the olfactory bulbs, the hippocampal formation, amygdala and the midbrain raphe nuclei. Immunohistochemical procedure was performed for SERT, BrdU, Ki-67, NeuroD, NeuN, Doublecortin (DCX), glial fibrillary acidic protein (GFAP) and Iba-1 using biotin-labeled antibody procedure.^17^ Details are shown in Supplementary Information.

### Confocal fluorescence microscopy

Intracellular C-NS-siRNA distribution in 5-HT neurons was examined by confocal microscopy using a Leica TCS SP5 laser scanning confocal microscope (Leica Microsystems Heidelberg GmbH, Manheim, Germany) equipped with a DMI6000 inverted microscope, blue diode (405 nm), Argon (458/476/488/496/514), diode-pumped solid state (561 nm) and HeNe (594/633nm) lasers. After i.n. administration with Alexa488 labeled C-NS-siRNA at 30 μg day^−1^ during 4 days, mice were killed and their brain were extracted and processed for immunofluorescence. Details are shown in Supplementary Information.

### Intracerebral microdialysis

Extracellular 5-HT concentration was measured by *in vivo* microdialysis as previously described.^17, 18, 25^ Briefly, one concentric dialysis probe (Cuprophan; 1.5-mm long) was implanted in caudate putamen (CPu; coordinates in mm: AP, 0.5; ML, −1.7; DV, −4.5) or ventral hippocampus (vHPC; AP, −3.0; ML, −3.0; DV, −4.0)^26^ of pentobarbital-anaesthetized mice. Experiments were performed 24–48h after surgery. To assess 8-OH-DPAT effects on extracellular 5-HT, 1 μM SSRI citalopram (Lundbeck A/S, Valby, Copenhagen, Denmark) was added to artificial cerebrospinal fluid. Artificial cerebrospinal fluid was pumped (WPI model, SP220i) at 2.0 μl min^−1^ and 30-min samples were collected. 5-HT concentrations were analyzed by high-performance liquid chromatography amperometric detection (+0.6V; Hewlett Packard 1049, Palo Alto, CA, USA) with 3-fmol detection limits. Baseline 5-HT levels were calculated as the average of four predrug samples.

### Behavioral studies

All mice were tested at 24h after treatments. All tests were performed between 1000 and 1500 hours. Behavioral assessments were examined with at least an interval of 1–2 days between tests. They were conducted in the following order: (1) open field test, (2) sucrose preference test, (3) novelty suppressed-feeding test, and (4) tail suspension test. On test days, animals were transported to a dimly illuminated behavioral room and were left undisturbed for at least 1 h before testing. Behavioral tests were conducted by an experimenter blind to mouse treatments. Details are shown in Supplementary Information.

### Statistical analyses

All results are given as mean±s.e.m. Data were analyzed using GraphPad Prism 6.0 (GraphPad, San Diego, CA, USA). Statistical analyses were performed by two-tailed Student's *t*-test and one-way or two-way analysis of variance followed by Tukey's *post-hoc* test as appropriate. In novelty suppressed-feeding test, we used the Kaplan–Meier survival analysis due to the lack of normal distribution of the data. Animals that did not eat during the 10-min testing period were discarded. Mantel–Cox log-rank test was used to evaluate differences between experimental groups, as described by Samuels and Hen.^27^ Differences were considered significant when *P*<0.05. Completed statistical analyses are summarized in Supplementary Table S3.

## Results

### Sertraline-conjugated siRNA is internalized into 5-HT neurons by endocytosis after i.n. administration

We used a previously developed strategy of transporter-mediated neuronal delivery of siRNA, in which the SSRI sertraline—which selectively binds to SERT—was chemically conjugated to the oligonucleotide.^18^ The working hypothesis was that the presence of sertraline would allow the selective enrichment of this conjugated siRNA in 5-HT neurons, where the targeted transporter (SERT) is differentially expressed.^28^ As first evidence in support of this mechanism, we previously showed that sertraline pretreatment (20 mg kg^−1^, i.p.) prevented the effects of sertraline-conjugated siRNA, indicating that conjugated siRNA molecules enter 5-HT neurons via SERT.^18^

**Figure 1:**
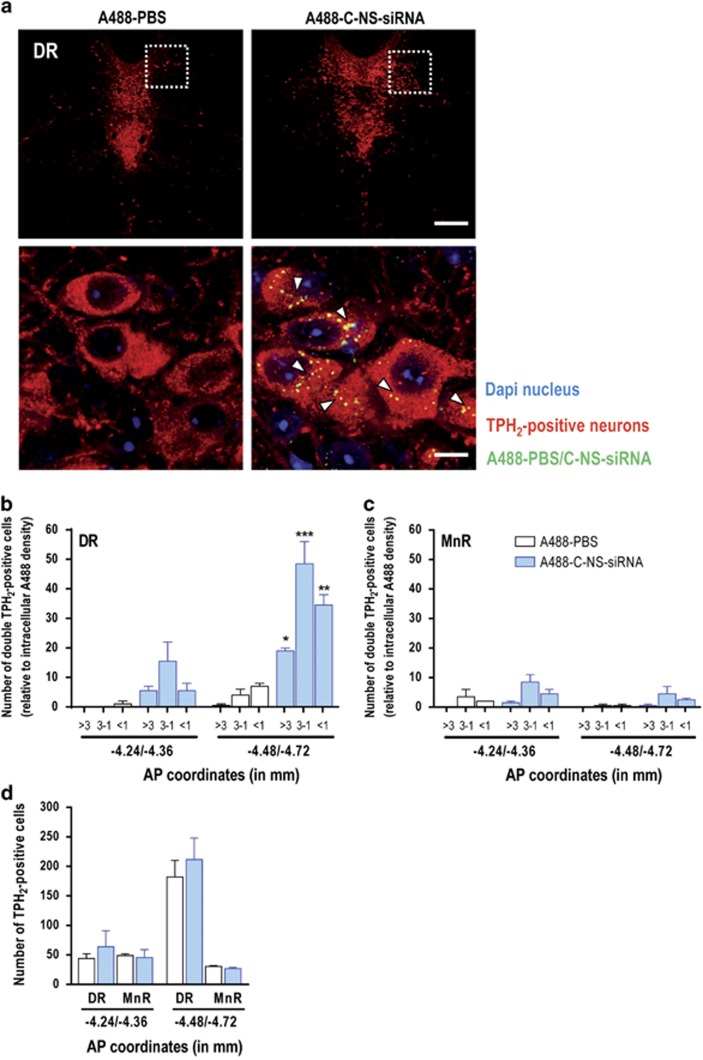
Selective accumulation of sertraline-conjugated nonsense-siRNA (C-NS-siRNA) in tryptophan hydroxylase2-positive (TPH_2_-positive) 5-hydroxytryptamine (5-HT) neurons after intranasal administration. Mice were intranasally administered with alexa488 phosphate-buffered saline (PBS) (A488-PBS) or alexa488-labeled C-NS-siRNA (A488-C-NS-siRNA) at 30 μg day^−1^ during 4 days and were killed 6 h postadministration (*n*=2 mice/group). (**a**) Confocal images showing co-localization of A488-C-NS-siRNA (yellow) in dorsal raphe nucleus (DR) 5-HT neurons (TPH_2_-positive, red) identified with white arrowheads. Cell nuclei were stained with DAPI (4,6-diamidino-2-phenylindole; blue). Bottom row are high-magnification photomicrographs of the frames in top row. Scale bars: low=200 μm, high=10 μm. (**b** and **c**) Histograms show the distribution profile of the abundance of A488-C-NS-siRNA (expressed as fluorescence units, ranges shown below the abscissa axis) in TPH_2_-positive neurons. Note the greater number of TPH_2_-positive cells co-localized with A488-C-NS-siRNA in the DR compared with median raphe nucleus (MnR). Range: >3, 3–1 and <1 represent relative unit of intracellular A488 density. (**d**) Number of TPH_2_-positive cells in the DR and MnR of mice. AP coordinates (in mm): −4.24/−4.36 and −4.48/−4.72 from bregma (*n*=2 mice/group). *P<0.05, **P<0.01, ***P<0.001 versus A488-PBS-treated mice. Data are mean±s.e.m.

For localization purposes, we synthesized an alexa488-labeled sertraline-conjugated nonsense-siRNA (A488-C-NS-siRNA). Confocal fluorescence microscopy revealed that A488-C-NS-siRNA was intracellularly detected in TPH_2_-positive midbrain 5-HT neurons after i.n. administration (Figure 1a). Confocal analysis showed that A488-C-NS-siRNA molecules were more efficiently uptaken by TPH_2_-positive neurons in the DR than in median raphe nucleus (MnR) (Figures 1b and d and Supplementary Figure S2). In addition, A488-C-NS-siRNA was absent in cells of brain areas close to the application site (olfactory bulbs) or to brain ventricles (hippocampus) (Supplementary Figure S3), supporting that surface SERT expression is a requirement for oligonucleotide uptake and internalization. Sertraline-conjugated siRNA was possibly accumulated in 5-HT cells perhaps by endocytosis and entered in a complex network of trafficking pathways as vesicles containing A488-C-NS-siRNA co-localized with Rab5 (early endosome marker) and Rab7 (late endosome marker) (Supplementary Figure S4). Further studies are needed to fully characterize the route used by sertraline-conjugated siRNA molecules to reach raphe 5-HT neurons.

### Sertraline-conjugated SERT-siRNA induces selective and safe suppression of SERT expression

**Figure 2:**
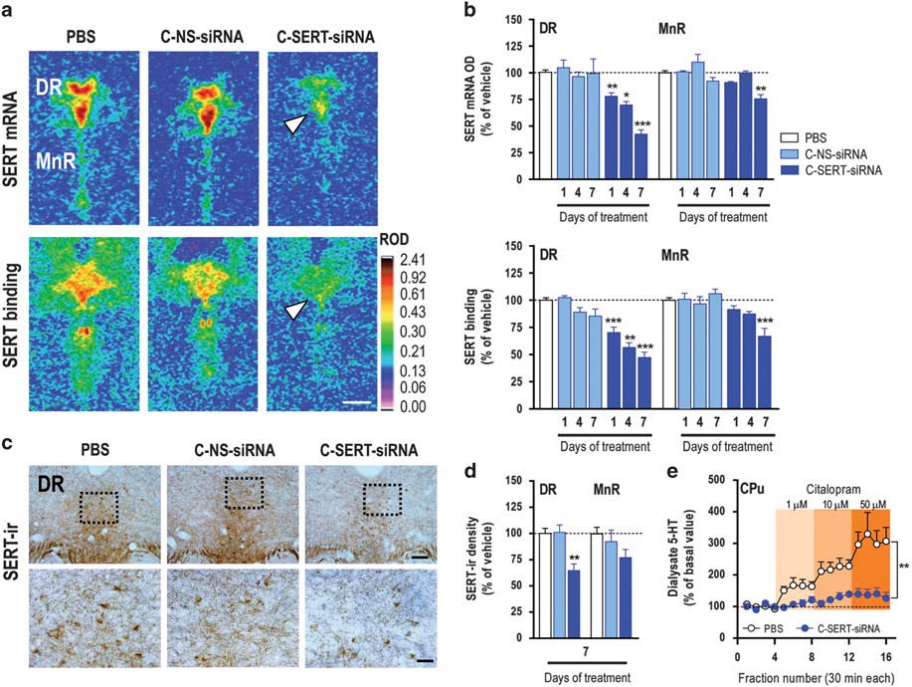
Intranasal sertraline-conjugated serotonin transporter small interfering RNA (SERT-siRNA) (C-SERT-siRNA) treatment downregulates SERT expression. Mice received intranasally: phosphate-buffered saline (PBS), sertraline-conjugated nonsense-siRNA (C-NS-siRNA) or C-SERT-siRNA at 30 μg day^−1^ (2.1 nmol day^−1^) during 1, 4 or 7 days. (**a**) Coronal brain sections showing reduced SERT mRNA and binding site levels in the dorsal raphe nucleus (DR) (AP coordinates: −4.48 to −4.72 in mm) of mice treated with C-SERT-siRNA (7-day). Scale bar: 500 μm. (**b**) Effects of C-SERT-siRNA on SERT mRNA and binding site densities in the DR and median raphe nucleus (MnR) (*n*=3–8 mice/group; *P<0.05, **P<0.01, ***P<0.001 versus PBS- and C-NS-siRNA-treated mice). (**c**) Immunohistochemistry images showing the expression of SERT protein (SERT-ir) in mouse DR. Bottom row are high-magnification photomicrographs of the frames in top row. Scale bars: low=100 μm, high=20 μm. (**d**) C-SERT-siRNA treatment (7-day) decreased DR SERT protein density versus PBS- and C-NS-siRNA-treated mice (*n*=3–5 mice/group; **P<0.01). (**e**) Local selective serotonin reuptake inhibitor citalopram infusion by reverse-dialysis induced concentration-dependent increases of extracellular 5-hydroxytryptamine (5-HT) in the caudate putamen (CPu) of PBS-treated mice more than in C-SERT-siRNA-treated mice (*n*=7–8 mice/group; **P<0.01 versus PBS). Data are mean±s.e.m.

We first examined the effect of i.n. C-SERT-siRNA administration (30 μg day^−1^) on SERT expression. SERT mRNA and binding site levels were significantly lower in the raphe nuclei of C-SERT-siRNA-treated mice compared with control groups (PBS- and C-NS-siRNA-treated mice), with a maximal reduction after 7-day treatment. Immunohistochemistry analysis confirmed these results at the protein level (Figures 2a and d and Supplementary Figure S5). C-SERT-siRNA suppressed SERT expression more markedly in the DR than in MnR, as supported by the higher intracellular density of oligonucleotide in DR 5-HT neurons (Figure 1). We then evaluated the dose-related effects of C-SERT-siRNA (10–30–100 μg day^−1^ during 7 days, i.n.) on SERT expression in the DR. All doses significantly decreased SERT mRNA expression 24 h after last administration, with no significant differences between doses, despite a slightly greater reduction at 30 and 100 μg day^−1^ (Supplementary Figure S6). Next we assessed the temporal pattern of SERT decrease. For this purpose, mice were i.n. administered with C-SERT-siRNA at 30 μg day^−1^ during 7 days and killed at different times after last administration. SERT mRNA level in the DR was significantly lower in C-SERT-siRNA-treated mice that in the control group at 1 and 3 days postadministration, with a recovery of SERT expression to control values at 7 and 15 days postadministration (Supplementary Figure S7). These values agree with data in the literature indicating a short half-life of SERT.^19^

The reduced SERT expression was accompanied by a decreased function, as assessed by intracerebral microdialysis in the CPu, a DR-innervated area. Hence, i.n. C-SERT-siRNA treatment (30 μg day^−1^, 7-day) doubled basal extracellular 5-HT levels in the CPu versus PBS-treated mice (9.9±1.6 and 4.7±0.7 fmol fraction^−1^, respectively; *n*=7-8; *P*<0.01) and provoked a lesser response of the SSRI citalopram to increase extracellular 5-HT (Figure 2e).

C-SERT-siRNA (30 μg day^−1^, 7-day, i.n.) did not induce neuronal degeneration (NeuN-positive), astrogliosis (GFAP-positive) nor immune responses (Iba-1-positive) (Supplementary Figure S8). Similarly, 7-day C-SERT-siRNA did not alter TPH_2_ mRNA levels in 5-HT neurons nor the binding density of [^3^H]-nisoxetine, which recognizes norepinephrine transporter (Supplementary Figure S9). Altogether, these data support the specificity and safety of C-SERT-siRNA effects.

### RNAi-based SERT suppression rapidly attenuates 5-HT_1A_-autoreceptor expression/function and enhances forebrain 5-HT transmission

**Figure 3:**
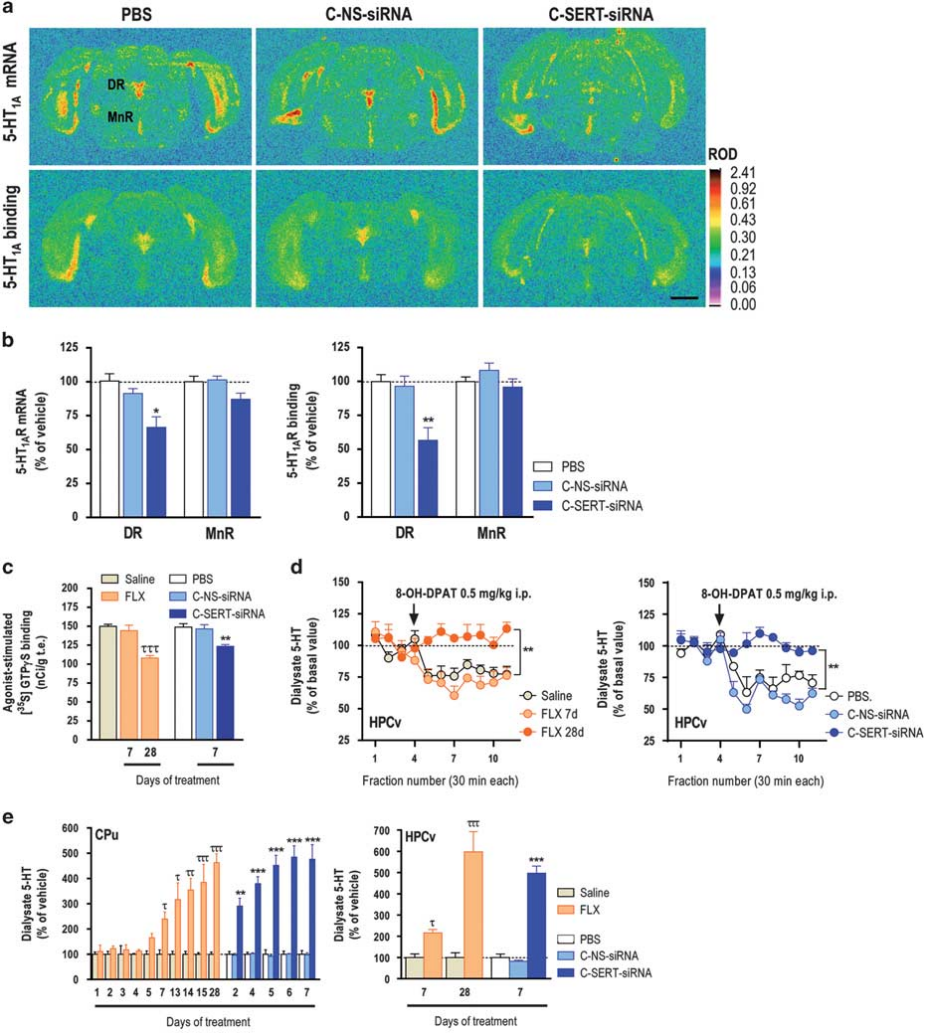
RNA interference-induced serotonin transporter (SERT) suppression reduces 5-hydroxytryptamine 1A (5-HT_1A_)-autoreceptor expression/function and rapidly enhances the forebrain 5-HT transmission. Mice were intranasally administered with: phosphate-buffered saline (PBS), sertraline-conjugated nonsense-small interfering RNA (siRNA) (C-NS-siRNA) or sertraline-conjugated SERT-siRNA (C-SERT-siRNA) at 30 μg day^−1^ during 7-day treatment. Other groups of mice were treated with saline or fluoxetine (FLX) at 10 mg kg^−1^ day^−1^, intraperitoneally during 7- or 28-day treatment. (**a**) Representative coronal brain sections showing reduced 5-HT_1A_ receptor mRNA and binding site levels in the dorsal raphe nucleus (DR) of C-SERT-siRNA-treated mice. Scale bar: 2 mm. (**b**) Effects of C-SERT-siRNA on 5-HT_1A_ receptor mRNA and binding site densities in the DR and median raphe nucleus (MnR) of mice (*n*=3–4 mice/group; *P<0.05, **P<0.01 compared with PBS- and C-NS-siRNA-treated mice). (**c**) Effects of C-SERT-siRNA and FLX on 5-HT_1A_-autoreceptor function. C-SERT-siRNA (7-day) decreased 5-HT_1A_ receptor-mediated 8-OH-DPAT-stimulated [^35^S]GTPγS binding in the DR, whereas FLX (7-day) was without effect (*n*=3–4 mice/group; **P<0.01 versus PBS- and C-NS-siRNA-treated mice). FLX reduced 5-HT_1A_-autoreceptor function after 28-day treatment (*n*=3–4 mice/group; ^τττ^*P*<0.001 versus saline and FLX 7-day). (**d**) 8-OH-DPAT did not reduce 5-HT release in the ventral hippocampus (HPCv) of C-SERT-siRNA-treated mice (7-day), unlike control groups (*n*=3–4 mice/group; **P<0.01 versus PBS and C-NS-siRNA). However, 8-OH-DPAT decreased hippocampal 5-HT concentration in saline- and FLX-treated 7-day mice but not in 28-day FLX-treated mice (*n*=5–8 mice/group; ^ττ^*P*<0.01 versus saline and FLX 7-day). (**e**) Intranasal C-SERT-siRNA treatment increased extracellular 5-HT levels in CPu more rapidly than FLX (*n*=4–10 mice/group; **P<0.01, ***P<0.001 versus PBS and C-NS-siRNA; ^τ^*P*<0.05, ^ττ^*P*<0.01, ^τττ^*P*<0.001 versus saline). Significant differences versus their respective control mice occurred after 2-day C-SERT-siRNA and after 7-day FLX treatment. Similar temporal differences were observed in HPCv (*n*=3-6 mice/group; ***P<0.001 versus PBS and C-NS-siRNA; ^τ^*P*<0.05, ^τττ^*P*<0.001 versus saline). Data are mean±s.e.m.

Concurrently, 7-day C-SERT-siRNA treatment reduced 5-HT_1A_-autoreceptor expression and function in mouse (Figures 3a and d), as also described after DR infusion of a unmodified SERT-siRNA in mouse or SERT antisense-plasmid in rat.^17, 19^ C-SERT-siRNA diminished 5-HT_1A_-autoreceptor mRNA level in the DR at both doses tested (30 and 100 μg day^−1^), but not 10 μg day^−1^, 24 h after last siRNA administration (Supplementary Figures S10a and b). Decreased 5-HT_1A_-autoreceptor response is necessary for the clinical antidepressant action, as 5-HT_1A_-autoreceptor activation by the excess 5-HT produced by SSRI/SNRI in the DR reduces 5-HT neuronal activity and 5-HT release, thus counteracting the facilitation of 5-HT transmission induced by SERT blockade.^29, 30, 31^ This inhibitory feedback is an essential component of the delayed therapeutic action of antidepressant drugs. Long-term FLX treatment (10 mg kg^−1^, 28-day, but not 7-day, i.p.) attenuated 5-HT_1A_-autoreceptor-mediated activation of G-proteins as expected^32^ and prevented the effect of 8-OH-DPAT (selective 5-HT_1A_ receptor agonist) to reduce hippocampal 5-HT release. In contrast, 7-day C-SERT-siRNA administration (30 μg day^−1^) was sufficient to downregulate 5-HT_1A_-autoreceptors and to avoid the 8-OH-DPAT effect on 5-HT release (Figures 3c and d). Neither treatment modified the hippocampal [^35^S]GTP-γ-S binding in the presence of 8-OH-DPAT (Supplementary Figure S10c). The reduction in SERT expression/function, together with the 5-HT_1A_-autoreceptor downregulation, increased extracellular 5-HT concentration in the CPu and hippocampus more rapidly and markedly than after FLX (Figure 3e). The present results indicate that cellular changes—classically associated to long-term SSRI treatment—can be achieved after only 1 week of i.n. C-SERT-siRNA administration. These observations suggest that SSRI and C-SERT-siRNA distinctly regulate the molecular mechanisms controlling SERT function, resulting in a more rapid and efficient increase of presynaptic 5-Ht function with the RNAi strategy.

### Sertraline-conjugated SERT-siRNA rapidly increases and facilitates maturation of newborn cells

**Figure 4:**
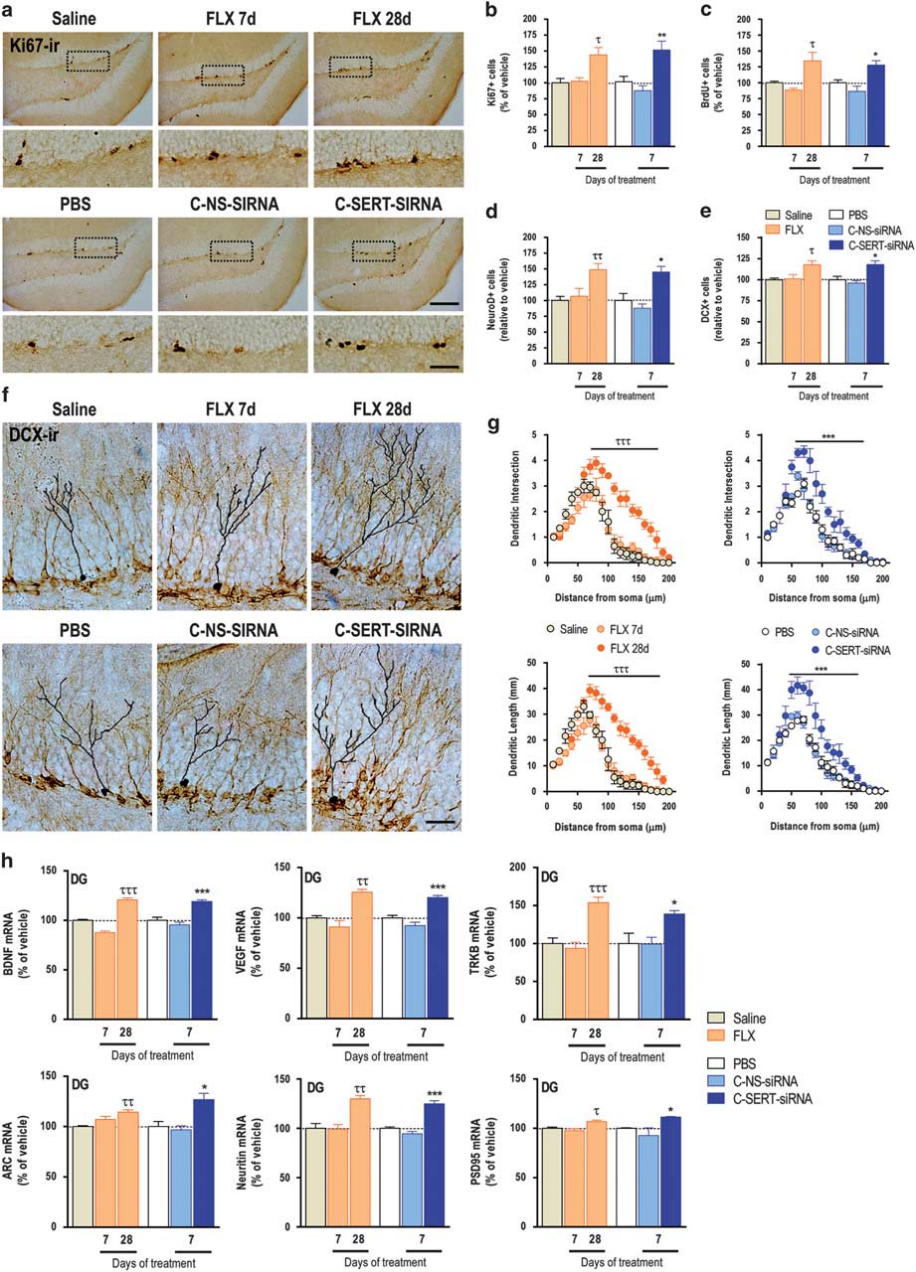
Intranasal administration of sertraline-conjugated serotonin transporter small interfering RNA (SERT-siRNA) (C-SERT-siRNA) accelerates the proliferation of cellular precursors and dendrite complexity in the hippocampus. (**a**) Representative images showing an increased number of Ki67-positive cells in the dentate gyrus (DG) of C-SERT-siRNA (7-day) or fluoxetine-treated mice (FLX, 28-day) versus their respective control mice. Bottom row shows high-magnification photomicrographs of the top row frames. Scale bars: low=100 μm and high=20 μm. (**b** and **c**) Short-term C-SERT-siRNA (7-day) or long-term FLX (28-day) treatments increased similarly the number of DG Ki-67-positive cells (*n*=5–10 mice/group) and 5-bromo-2'-deoxyuridine (BrdU)-positive cells (*n*=6–10 mice/group). *P<0.05, **P<0.01 versus phosphate-buffered saline (PBS) and sertaline-conjugated nonsense-siRNA (C-NS-siRNA); ^τ^*P*<0.05 versus saline and FLX 7-day treatment. (**d** and **e**) Short-term C-SERT-siRNA treatment or chronic FLX administration increased similarly the number of immature neurons identified with NeuroD (*n*=4–10 mice/group) or doublecortin (DCX; *n*=5-11 mice/group) markers (*P<0.05 versus PBS and C-NS-siRNA; ^τ^*P*<0.05, ^ττ^*P*<0.01 versus saline and FLX 7-day treatment). (**f**) Representative images and traces from Sholl analyses of DCX-positive cells bearing a complex dendritic morphology in the DG of mice in the different treatment group. Scale bar: 20 μm. (**g**) Effects of C-SERT-siRNA (7-day) or FLX (28-day) treatments on dendritic intersection numbers and dendritic length of DCX-positive neurons (*n*=4 mice/group, 5 cells/mouse; ***P<0.001 versus PBS and C-NS-siRNA; ^τττ^*P*<0.001 versus saline and FLX 7-day). (**h**) Levels of mRNA for the following genes: BDNF, VEGF, TRKB, ARC, Neuritin, and PSD95 in the DG were analyzed by densitometry and are shown in the bar graphs (*n*=3–10 mice/group; *P<0.05, ***P<0.001 versus PBS and C-NS-siRNA; ^τ^*P*<0.05, ^ττ^*P*<0.01, ^τττ^*P*<0.001 versus saline and FLX 7-day treatment). Values are mean±s.e.m.

The clinical antidepressant action is also associated with neural stem cell proliferation, neurogenesis and the establishment of new synaptic contacts in brain circuits controlling motivation, emotion and cognition.^33, 34^ Treatments with C-SERT-siRNA (30 μg day^−1^, 7-day) or FLX (10 mg kg^−1^, 28-day, but not 7-day) markedly increased the number of Ki67- and BrdU-labeled progenitor cells in the dentate gyrus of hippocampus (Figures 4a–c, Supplementary Figure S11a, Supplementary Table S4). In addition, 7-day C-SERT-siRNA promoted significantly the generation of NeuroD- and DCX-expressing neurons faster than FLX (28-day) (Figures 4d and e, Supplementary Figure S11b, Supplementary Table S4). Dendritic morphology of newborn cells was comparable after 7-day C-SERT-siRNA and 28-day FLX treatments, as indicated by Sholl analysis on DCX-positive neurons with tertiary dendrites, the number of intersections and dendrite length (Figures 4f and g). These data support that RNAi-induced SERT knockdown rapidly enhances neuronal plasticity in hippocampus as a result of the increased 5-HT signaling.

These cellular changes were accompanied by a higher activation of neuroplasticity-associated genes in the hippocampus. Seven-day C-SERT-siRNA or 28-day FLX treatments increased comparably the expression of BDNF and its TRKB receptor as well as VEGF and ARC essentially in the dentate gyrus (Figure 4h and Supplementary Figure S12). Likewise, mRNA neuritin and PSD95 levels, two critical downstream mediators of antidepressant/BDNF-induced plasticity^35, 36, 37^ were selectively increased in the dentate gyrus of both the mice groups. However, a 7-day FLX regime was without effect on these variables (Figure 4).

### Short-term sertraline-conjugated SERT-siRNA efficiently attenuates the behavioral alterations in the corticosterone depression model

**Figure 5:**
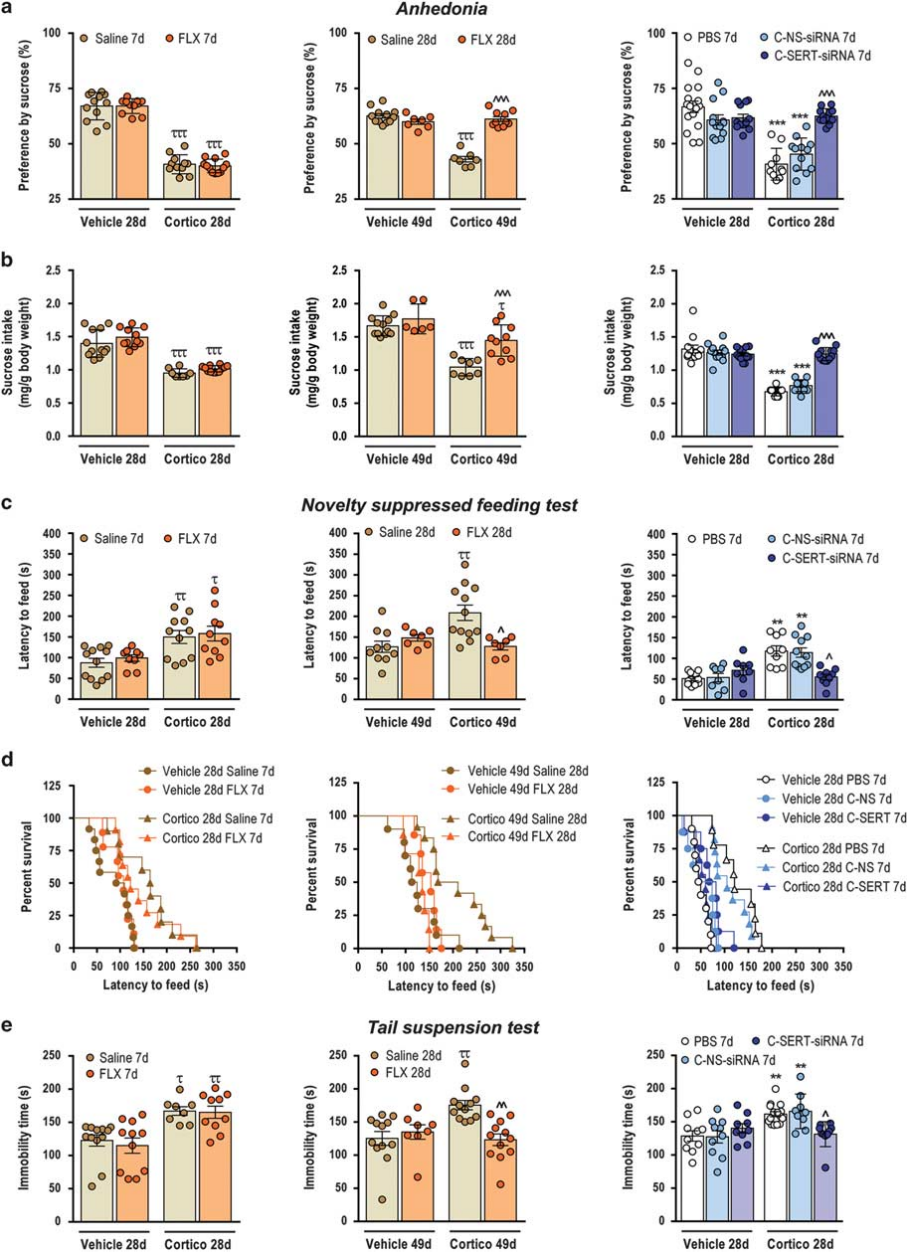
Short-term intranasal (i.n.) treatment with sertraline-conjugated serotonin transporter small interfering RNA (SERT-siRNA) (C-SERT-siRNA) efficiently attenuates the behavioral deficits in a stress-induced depression model. Grouped-housed male C57BL/6J mice were presented during 28 or 49 days with vehicle (non-stressed mice) or corticosterone (stressed mice) in the presence or absence of an antidepressant treatment (C-SERT-siRNA 30 μg day^−1^, i.n. or fluoxetine (FLX) 10 mg kg^−1^ day^−1^, intraperitoneally) during the last 7 or 28 days of the corticosterone regimen. (**a** and **b**) Short-term C-SERT-siRNA reversed the reduction of sucrose intake and preference in the corticosterone-induced anhedonia (*n*=10–16 mice/group; ***P<0.001 versus non-stressed mice; ^^^*P*<0.001 versus corticosterone-stressed mice treated with phosphate-buffered saline (PBS) or sertraline-conjugated nonsense-siRNA (C-NS-siRNA)). FLX induced a similar recovery after 28-day, but not after 7-day, treatment (*n*=7–12 mice/group; ^τττ^*P* <0.001 versus non-stressed mice; ^^^*P*<0.001 versus corticosterone-stressed mice treated with saline. (**c**) Effect on novelty suppressed feeding test (NSFT). Seven-day C-SERT-siRNA, but not 7-day FLX, reversed the increased latency to feed in corticosterone-treated mice (*n*=8–12 mice/group; **P<0.01 versus non-stressed mice; ^*P*<0.05 versus corticosterone-stressed mice treated with PBS or C-NS-siRNA). Similar effects were elicited by 28-day FLX administration (*n*=7–12 mice/group; ^ττ^*P*<0.01 versus non-stressed mice; ^*P*<0.05 versus corticosterone-stressed mice treated with saline). (**d**) Survival analysis of NSFT data. (**e**) C-SERT-siRNA (7-day) or FLX (28-day), but not FLX 7-day, decreased the immobility time in the tail suspension test (TST) in cortico-stressed mice (*n*=8–15 mice/group). **P<0.01, ***P<0.001, ^τ^*P*<0.05, ^ττ^*P*<0.01 versus their non-stressed mice, respectively; ^*P*<0.05, ^^*P*<0.01 versus cortico-stressed mice treated with saline, PBS or C-NS-siRNA, respectively. Values are mean±s.e.m.

Finally, we confirmed the potential therapeutic benefit of the RNAi-induced downregulation of SERT expression in a stress-related model of depression: the corticosterone model—a well-established stress inducer.^12, 22^ Despite displaying plasma corticosterone levels and open field behavior similar to controls, mice exposed to a low oral dosage of corticosterone for 28 or 49 days showed a persistent depressive-like behavior, characterized by reduced sucrose preference, increased latency in the novelty suppressed feeding paradigm and increased immobility time in the tail suspension test (Supplementary Figure S13). These depressive-like behaviors were reversed to the same extent by 7-day i.n. C-SERT-siRNA (30 μg day^−1^) and by 28-day FLX (10 mg kg^−1^) treatments (Figure 5). In contrast, 7-day FLX did not evoke any antidepressant-like effects.

## Discussion

Here we show that C-SERT-siRNA evokes very fast (7-day) and robust antidepressant-like responses in control and corticosterone-treated mice, comparable than those evoked by a 28-day FLX treatment. Prior studies using RNAi strategies to elicit antidepressant-like responses in rodents used unmodified siRNA sequences directed against SERT or 5-HT_1A_ receptors.^16, 17, 25^ The study confirms and extends our previous observations on the use of sertraline-conjugated siRNA sequences to silence genes in 5-HT neurons.^18^ The design of the conjugated siRNA allows its selective enrichment in 5-HT neurons after i.n. administration, opening new ways for the therapeutic use of RNAi strategies to treat mood disorders, which may overcome the limitations of standard antidepressant treatments, that is, slow clinical action and low efficacy.

As a consequence of the fast and effective SERT downregulation, 7-day C-SERT-siRNA treatment (i) reduced 5-HT_1A_-autoreceptor expression/function, (ii) facilitated forebrain serotonin neurotransmission, (iii) accelerated the proliferation of neuronal precursors, (iv) increased the expression of growth factors (for example, BDNF, VEGF) and genes promoting neurite outgrowth (for example, neuritin, PSD95) and (v) increased hippocampal dendritic complexity and synaptic plasticity. All these variables are predictive of clinical antidepressant action. In addition, short-term C-SERT-siRNA treatment normalized stress-induced depressive-like behaviors, which were only sensitive to 28-day FLX treatment. Interestingly, the above actions were produced by the i.n. administration of very small doses (2.1 nmol day^−1^) of C-SERT-siRNA illustrating the effectiveness of the present RNAi strategy.

The precise mechanism(s) used by C-SERT-siRNA reach 5-HT neurons are not fully understood. Unlike other strategies used for oligonucleotide delivery to target neuronal populations,^38, 39^ here we used a transporter (SERT)-mediated process to target a selective gene (SERT) expressed in 5-HT neurons, as previously used to silence 5-HT_1A_-autoreceptors.^18^ Paradoxically, this approach could limit the extent of the intracellular C-SERT-siRNA accumulation, as the conjugated siRNA enters 5-HT neurons via SERT.^18^ However, the present results indicate that the remaining expression of SERT—even after 7-day C-SERT-siRNA treatment—is sufficient to evoke a very large increase of presynaptic serotonergic function.

Moreover, as trans-nasal oligonucleotide delivery to brain is mediated by extracellular mechanisms,^40, 41, 42^ it is also possible that C-SERT-siRNA can use this extracellular pathway before being taken up by serotonergic terminals and transported back to cell bodies in the midbrain. This view is showed by the association of the conjugated siRNA to Rab7, supporting traffic via late endomembrane compartments.

The present results indicate that C-SERT-siRNA is preferentially accumulated into DR (versus MnR) 5-HT neurons. Anatomical and functional differences between the two 5-HT subsystems have been reported.^43, 44, 45^ In particular, SERT expression is greater in DR than in MnR and DR-innervated areas are more sensitive to the action of SSRI,^40^ which suggests a preferential uptake of C-SERT-siRNA by DR axons.

Consistent with previous findings using intra-raphe SERT-siRNA infusion,^17^ i.n. C-SERT-siRNA treatment triggered a complex cascade of signaling events that ultimately results in downregulation of SERT and also 5-HT_1A_-autoreceptors on midbrain serotonin neurons. As both mechanisms tightly control the active 5-HT fraction, their downregulation by C-SERT-siRNA dramatically increased the extracellular 5-HT levels in the forebrain in a faster way than that produced by the pharmacological SERT blockade with FLX. The intracellular mechanisms by which short-term C-SERT-siRNA or chronic SSRI treatments downregulate SERT and 5-HT_1A_ receptor levels to increase the serotonergic neurotransmission are still poorly known. Recent reports suggest a role for altered patterns of gene expression in mediating the long-term therapeutic effects of SSRIs, focusing on the potential involvement of microRNAs as fine-tuners and on–off switches of gene expression.^15, 46^ Hence, miR-135 levels were upregulated after single and chronic FLX administrations,^47^ suggesting that this microRNA may act as an endogenous homeostatic mechanism to maintain the physiological balance between SERT and 5-HT_1A_-autoreceptors.

Similarly to intra-raphe SERT-siRNA application,^17^ the short-term i.n. administration of C-SERT-siRNA—but not FLX—evoked hippocampal postsynaptic responses to the enhanced serotonergic signaling, which are predictive of clinical antidepressant effects. These responses included the proliferation of progenitor cells, stimulation of dendritic branching, acceleration of the maturation of immature DCX neurons and increased expression of spine/synapse markers. Moreover, these postsynaptic changes were accompanied by the reversal of the behavioral deficits caused by prolonged corticosterone exposure. The C-SERT-siRNA ability to increase synaptic plasticity may be mediated by an enhancement of BDNF/TRKB signaling, among others that were upregulated after 7-day C-SERT-siRNA treatment. Activation of their downstream signaling pathways was shown to enhance maturation and dendritic development.^48^ Induction of neuritin and PSD95 are also indicative of an increased synapse formation and function.^37^ However, additional antidepressant mechanisms may be involved, resulting from the enhanced 5-HT neurotransmission in other brain areas implicated in MDD, such as the ventromedial prefrontal cortex.

In conclusion, our findings indicate that RNAi-based SERT repression elicits faster and more effective antidepressant-like actions than persistent SERT blockade with FLX. Enhancing serotonin signaling through downregulation of SERT expression evokes standard antidepressant responses, promotes the generation of new hippocampal neurons, increases synaptic plasticity and counteracts behavioral deficits in a stress-induced depression model. Furthermore, the study has a high translational value due to the use of a clinically feasible (i.n.) administration route.^40, 41, 42^ This opens new therapeutic perspectives for the treatment of mood disorders, including MDD.
