# Supplementary material for: Therapeutic antidepressant potential of a conjugated siRNA silencing the serotonin transporter after intranasal administration
Source: Mol Psychiatry. 2015 Jun 23;21(3):328–38. doi: 10.1038/mp.2015.80 (PMC4759205; doi:10.1038/mp.2015.80)
Supplement: Supplementary Information [file mp201580x2.doc]

**Supplementary Information for**

**Therapeutic antidepressant potential of a conjugated siRNA silencing the serotonin transporter after intranasal administration**

Albert Ferrés-Coy^1,2,3^, Mireia Galofré^1,2,3^, Fuencisla Pilar-Cuéllar^3,4^, Rebeca Vidal^3,4^, Verónica Paz^1,2,3^, Esther Ruiz-Bronchal^1,2,3^, Letizia Campa^1,2,3^, Ángel Pazos^3,4^, Javier R Caso^3,5^, Juan C Leza^3,5^, Gabriel Alvarado^6^, Andrés Montefeltro^6^, Elsa M Valdizán^3,4^, Francesc Artigas^1,2,3^ and Analia Bortolozzi^1,2,3*^

**The PDF file includes:**

Materials and Methods

References

Supplementary Figures S1 – S13

Supplementary Table S1 – S4

**Materials and Methods**

**Conjugated siRNA synthesis**

The synthesis and purification of sertraline-conjugated siRNA directed against serotonin transporter-[SERT] (C-SERT-siRNA, nt: 1230–1250, GenBank accession NM_010484) and sertraline-conjugated nonsense-siRNA (C-NS-siRNA) molecules were performed by nLife Therapeutics S.L. (Granada, Spain) as previously reported.^18^ In brief, siRNA (sense and antisense strands) synthesis was performed using ultra mild-protected phosphoramidites (Glen Research, Sterling, VA, USA) and H-8 DNA/RNA Automatic synthesizer (K&A Laborgeraete GbR, Schaafheim, Germany). Sense strand was amino-modified by performing a 5'-C6 amino modification and condensation with a succinimide active ester of sertraline (sertraline-NH-CH2C(=O)NH(CH2)5COO-succinimide) making up the peptide linker of the conjugate (sertraline-NH-CH2C(=O)NH(CH2)5COO-sense strand-3'OH). Conjugated single strand oligonucleotides were purified by high performance liquid chromatography using a RP-C18 column (4.6x150 mm, 5 µm) under a linear gradient condition of acetonitrile shifting the concentration from 5% to 35% for 30min in 100mM TEAA (pH 7.0). The molecular weights of the siRNA strands and the conjugate were confirmed by MALDI-TOF mass spectrometry (Ultraflex, Bruker Daltonics). The yield of the conjugates was spectrophotometrically calculated on the basis of absorbance at 260nm wavelength. Complementary strands were annealed in an isotonic RNA-annealing buffer (100mM potassium acetate, 30mM HEPES pH 7.4, 2mM magnesium acetate), pre-incubated by 1min at 90ºC, centrifuged for 15s and incubated 1h at 37ºC. Duplex RNA formation was confirmed using 20% polyacrylamide gel electrophoresis (PAGE, 30mA, 60min) and visualized by silver staining (DNA Silver stain kit, GE Healthcare, Piscataway, NJ). Sequences are shown in **Supplementary** **Table S1**.

**In situ hybridization**

Frozen tissue sections were first brought to room temperature, fixed for 20min at 4ºC in 4% paraformaldehyde in phosphate-buffered saline (1xPBS: 8mM Na_2_HPO_4_, 1.4mM KH_2_PO_4_, 136mM NaCl, and 2.6mM KCl), washed for 5min in 3xPBS at room temperature, twice for 5min each in 1xPBS, and incubated for 2min at 21ºC in a solution of predigested pronase (Calbiochem, San Diego, CA) at a final concentration of 24 U/mL in 50mM Tris-HCl, pH 7.5, and 5mM EDTA. The enzymatic activity was stopped by immersion for 30s in 2 mg/ml glycine in 1xPBS. Tissues were finally rinsed in 1xPBS and dehydrated through a graded series of ethanol. For hybridization, the radioactively labeled probes were diluted in a solution containing 50% formamide, 4x standard saline citrate, 1x Denhardt’s solution, 10% dextran sulfate, 1% sarkosyl, 20mM phosphate buffer, pH 7.0, 250 μg·ml^-1^ yeast tRNA, and 500 μg·ml^-1^ salmon sperm DNA. The final concentrations of radioactive probes in the hybridization buffer were in the same range (~1.5 nM). Tissue sections were covered with hybridization solution containing the labeled probes, overlaid with Nescofilm coverslips (Bando Chemical Ind., Kobe, Japan), and incubated overnight at 42ºC in humid boxes. Sections were then washed 4 times (45min each) in a buffer containing 0.6M NaCl and 10mM Tris-HCl (pH 7.5) at 60ºC. Hybridized sections were exposed to Biomax-MR film (Kodak, Sigma-Aldrich, Madrid, Spain) for 1-4 weeks with intensifying screens. For specificity control, adjacent sections were incubated with an excess (50x) of unlabelled probes. The cytoarchitecture of different mouse brain areas were analyzed in an adjacent series of cresyl-violet stained frozen sections.

**Autoradiographic studies**

The autoradiographic binding assays for 5-HT_1A_R, SERT and norepinephrine transporter (NET) were performed using the following radioligands: (a) [^3^H]-8-OH-DPAT (233 Ci·mmol^-1^), (b) [^3^H]-citalopram (70 Ci·mmol^-1^) and, (c) [^3^H]-nisoxetine (85 Ci·mmol^-1^), respectively (Perkin-Elmer, Madrid, Spain) as described previously.^17^ 8-OH-DPAT, isoproterenol, mazindol, pargyline and serotonin were from Sigma-Aldrich. The experimental conditions for incubation with each radioligand are summarized in **Supplementary** **Table S2**. After incubation and washes, tissues were dipped in distilled ice-cold water and dried rapidly under a cold air stream. Tissues were exposed to Biomax-MR film together with ^3^H-Microscales standards (Amersham-GE Healthcare, Barcelona, Spain) at 4ºC for 1-3 month. All experimental and control brains within a group were processed in duplicate and exposed to films as a batch.

For 5-HT_1A_R-stimulated [^35^S]GTPγS autoradiography, coronal dorsal raphe nucleus (DR) sections were pre-incubated for 30min at 25ºC in a buffer containing 50mM Tris–HCl, 0.2mM EGTA, 3mM MgCl_2_, 100mM NaCl, 1mM DTT and 2mM GDP (pH 7.7). Slides were subsequently incubated for 2h in the same buffer containing 10mU·ml^-1^ adenosine deaminase with 0.04nM [^35^S]GTPγS and consecutive sections were incubated with 10^-5^M 8-OHDPAT alone or in the presence of 10^-5^M WAY-100,635. Nonspecific binding was determined in the presence of 10μM GTPγS. Sections were exposed to autoradiographic film (Biomax-MR) together with ^14^C-polymer standards at 4ºC for two days.^17^

**Immunohistochemistry**

For SERT staining, brain sections were treated with 1x PBS, 100% methanol and 30% H_2_O_2_ for 30min. After incubation in 3% normal goat serum (Vector Laboratories, Burlingame, CA) in 0.2% PBS/Triton for 120min, the sections were incubated for 4 days in rabbit anti-SERT (1:2500; ref.: 24330 ImmunoStar, Inc, Hudson, WI) at 4°C. After washing with PBS, sections were incubated in biotinylated goat anti-rabbit IgG (1:200; ref.: BA-1000 Vector Laboratories) for 1h. After incubation with 1% avidin biotin complex (Vectastain Elite ABC Kit, Vector laboratories) for 1h, sections were washed and reacted for visualization using diaminobenzidine tetrahydrochloride (DAB) solution in a peroxidase reaction to produce a brown reaction product. Sections were mounted in Entellan (Electron Microscopy Sciences). Images were captured at a magnification of 20x by using a Nikon Eclipse E1000 microscope (Nikon, Tokyo, Japan) equipped with bright- and dark-field condensers for transmitted light and a digital camera (DXM1200 3.0, ACT-1 software, Nikon). For each mouse, measurements were taken from five consecutive coronal brain sections and, two areas per section were delineated using ImageJ Software. Optical density was obtained as the logarithmic ratio between SERT-ir intensity in dorsal raphe nucleus, hippocampus or amygdala and an adjacent area as background.

BrdU staining was performed as previously described.^17^ Free floating coronal hippocampus sections were incubated for 2h in 50% formamide/2x SSC at 65°C, followed by incubation in 2N HCl for 30min. Then sections were incubated for 10min in 0.1M borate buffer. After washing in PBS, sections were incubated in 1% H_2_O_2_ in PBS for 30min to inactive endogenous peroxidase activity. After several rinses in PBS, sections were incubated in PBS/0.2% Triton X-100/5% goat serum (PBS–TS) for 30min and then incubated with monoclonal mouse anti-BrdU (1:600; ref.: 11170376001 Roche Diagnostics, Barcelona, Spain) overnight at 4°C. After several rinses in PBS–TS, sections were incubated for 2h with biotinylated goat anti-mouse Fab Fragment IgG secondary antibody (1:200; ref.: 115-066-006 Jackson ImmunoResearch Laboratories, Inc., US-PA), followed by amplification with avidin–biotin complex (Vector Laboratories). For quantification of BrdU^+^ cells, one every sixth section throughout the hippocampus was processed and counted under a light microscope (Carl Zeiss Axioskop 2 Plus) at 40x and 100x magnification. The total number of BrdU^+^ cells per section were determined and multiplied by 6 to obtain the total number of BrdU^+^ cells per hippocampus.

Ki-67 staining was carried out in sections adjacent to those used in the BrdU labeling studies. Sections were prepared as above including the inactivation of endogenous peroxidase activity, washing in PBS and retrieving of the antigen by heating at 80ºC in 10 mM citric acid, pH 9.0 for 30min. The sample were then incubated with rabbit anti-Ki-67 (1:5000, ref.: ab16667 Abcam, Cambridge, UK) overnight at 4°C, washed with PBS, incubated with biotinylated donkey anti-rabbit secondary antibody (1:200; ref.: 711-065-152 Jackcon ImmunoResearch Laboratories, Inc., US) for 2h, and the signal was amplified with avidin–biotin complex (Vector Laboratories). Ki-67^+^ cells were labeled using DAB as chromogen (Vector Laboratories). The quantification of Ki67^+^ cells was performed as described for BrdU^+^ cells.

Goat anti-NeuroD (1:200; sc-1084, Santa Cruz Biotechnology, Santa Cruz, CA) and goat anti-Doublecortin (DCX; 1:200; ref.: sc-8066, Santa Cruz Biotechnology) were used as neurogenesis markers. After endogenous peroxidase inhibition and washes, pre-incubation and incubation were carried out in a 1x PBS/Triton 0.2% solution containing 10% and 3% normal donkey serum (Millipore), respectively. Primary antibodies were incubated overnight at 4ºC, followed by incubation with biotinylated donkey anti-goat (1:200; ref.: sc-2042, Santa Cruz Biotechnology), and subsequent incubation in ABC solution (Vector Laboratories) according to the manufacturer’s instructions. The color reaction was performed by incubation with DAB solution. The sections were mounted onto gelatin-coated slides, embedded and investigated on a Nikon Eclipse E1000 microscope (Nikon, Tokyo, Japan) using 20x and 40x objectives. Labeled cells (NeuroD^+^ or DCX^+^ neurons) were counting in three consecutive sections containing the hippocampal structures and six different microscope fields were analyzed in each section. In addition, DCX^+^ cells were subcategorized according to their dendritic morphology. Four randomly selected DCX^+^ cells per mouse (a total of 120 neurons) with tertiary relatively un-truncated dendritic branches were traced using NeuronJ plugin v1.4.2. Dendritic length and number of intersections (branch point) were evaluated using ImageJ (v1.47n) Software and Sholl Analysis plugin (v1.0).

For immunohistochemical identification of neurons (NeuN; 1:1000; ref.: MAB377, Merck Millipore, Madrid, Spain), reactive astrocytes (GFAP; 1:1000; ref.: Z0334, DAKO, Barcelona, Spain), or microglia (Iba-1; 1:1,000; ref.: 019-197741, WAKO, Irvine, CA), we used a biotin-labeled antibody procedure. Following endogenous peroxidase inhibition and washes, tissues were blocked for 2h in 3% normal goat serum, and primary antibody incubations were carried out overnight at 4ºC. Sections were incubated in goat anti-rabbit (1:200; ref.: BA-1000, Vector Laboratories, Burlingame, CA) or goat anti-mouse (1:200; ref.: sc-2039, Santa Cruz Biotechnology) biotinylated IgG secondary antibodies for 1h at room temperature and subsequent incubation in ABC solution. Sections were mounted onto gelatin-coated slides with Entelan. Images were captured by using a Nikon Eclipse E1000 microscope (Nikon, Tokyo, Japan) equipped with a digital camera (DXM1200 3.0, Nikon) and ACT-1 software (Nikon). Figures were assembled in Adobe Photoshop (Adobe Systems, San Jose, CA); only contrast and brightness were adjusted to optimize the images.

**Confocal fluorescence microscopy**

Brain sections were rinsed with PBS/Triton 0.2%, incubated with 10% normal serum from secondary antibody host and treated with primary antibodies: sheep anti-TPH_2_ (1:2500; ref.: AB1541, Merck Millipore), rabbit anti-Rab5 (1:500; ref.: ab18211, Abcam) or mouse anti-Rab7 (1:2000; ref.: R8779-200UL, Sigma-Aldrich). Sections were then incubated at 4ºC overnight, rinsed and treated with respectively secondary Alexa555-conjugated antibodies (1:500, A-20000, Life Technologies) for 120min. After subsequent washes, the section were dehydrated and mounted in the anti-fading agent Prolong Gold with DAPI (Life Technologies). DAPI, Alexa488 and Alexa555 images were acquired sequentially using 405, 488 and 561 laser lines, AOBS (Acoustic Optical Beam Splitter) as beam splitter and emission detection ranges 415- 480, 500-550 and 571-625 nm, respectively and, the confocal pinhole set at 1 Airy units. Images were acquired at 400Hz in a 1024 x 1024 pixels format. Images were composed using NIH ImageJ 1.49g software ((<http://rsbweb.nih.gov/ij/>). Images were acquired from two sections containing both DR and MnR at AP coordinates (in mm) of -4.24/-4.36 and -4.48/-4.72 from bregma for each mouse. For co-localization analysis, the confocal images were processed with the co-localization highlighter plugin, and only TPH_2_-positive cells with a threshold of more than 10 pixels co-localizing with A488-C-NS-siRNA or A488-PBS were taken into account.

**Behavioral studies**

*Open field.* This test was performed as described previously.^49^ Briefly, motor activity was measured in four Plexiglas open field boxes 35x35x40 cm indirectly illuminated (25-40 luxes) to avoid reflection and shadows. The floor of the open field was covered with an interchangeable opaque plastic base that was replaced for each animal. Motor activity was recorded during 15min by a camera connected to a computer (Videotrack, View Point, Lyon France). We automatically measured the following variables: horizontal locomotor and exploratory activity, defined as the total distance moved in cm including fast/large (speed > 10.5 cm·s^-1^) and slow/short movements (speed 3-10.5 cm·s^-1^), and the activation time, i.e., the time (sec) spent in movement.

*Sucrose preference test.* During this test, mice were given a free choice between two bottles, one with 1% sucrose solution and another with regular water, for 8h between 09:00-17:00h. To prevent the possible effects of a side-preference in drinking behavior, the position of the bottles in the cage was switched after 4h during the test. No previous food or water deprivation was applied before the test. The consumption of water and sucrose solution was assessed simultaneously in control and experimental groups by weighing the bottles. The sucrose intake was calculated as an amount of consumed sucrose in mg per gram body weight. The preference for sucrose was calculated as a percentage of consumed sucrose solution of the total amount of liquid drunk. A decrease of sucrose preference below 55%, measured at 28 days of chronic CORT exposure, was taken as the criterion for anhedonia.^50^

*Novelty suppressed feeding.* NSF is a conflict test that elicits competing motivations: the drive to eat and the fear of venturing into the center of the brightly lit arena. The NSF test was carried out during a 10 min period as previously described.^7,8^ The testing apparatus consisted of a plastic box (35x35x20 cm), the floor of which was covered with approximately 2 cm of wooden bedding for each animal. 24h prior to behavioral testing, all food was removed from the home cage. At the time of testing, a single pellet of food was placed on a white paper platform in the center of the box directly illuminated with 1100 luxes. An animal was placed in a corner of the box, and a stopwatch was immediately started. The latency to eat (defined as the mouse sitting on its haunches and biting the pellet with the use of forepaws) was timed. Immediately afterwards, the animal was transferred to its home cage and, the latency to feed and the amount of food consumed by the mouse in the subsequent 5 min was measured. Each mouse was weighed before food deprivation and before testing to assess the percentage of body weight loss.

*Tail suspension test.* Mice were suspended 30 cm above the bench by adhesive tape placed approximately 1 cm from the tip of the tail. The total duration of immobility during a 6min test was measured.^17,18,25^

**REFERENCES**

1. Scorza MC, Castañé A, Bortolozzi A, Artigas F. Clozapine does not require 5-HT1A receptors to block the locomotor hyperactivity induced by MK-801 Clz and MK-801 in KO1A mice. *Neuropharmacol.* 2010; **59**: 112-120.
2. Strekalova T, Spanagel R, Bartsch D, Henn FA, Gass P. Stress-induced anhedonia in mice is associated with deficits in forced swimming and exploration. *Neuropsychopharmacol.* 2004; **29**: 2007–2017.

**Supplementary Figures**

**Figure S1** Experimental timeline of corticosterone exposure and antidepressant treatments. Group-housed male C57BL/6J mice were presented during 28 or 49 days with vehicle or corticosterone at: 30 μg·ml^-1^ for 15 days, followed by 15 μg·ml^-1^ for 3 days and 7.5 μg·ml^-1^ for 10 or 31 days in the presence or absence of an antidepressant treatment (sertraline-conjugated SERT-siRNA -C-SERT-siRNA- 30 μg·day^-1^, i.n. or fluoxetine -FLX- 10 mg·kg^-1^·day^-1^, i.p.) during the last 7 or 28 days of the corticosterone regimen. We investigated whether the behavioral changes resulting from chronic corticosterone exposure were reversed by treatment with C-SERT-siRNA or FLX. The same animal was successively tested in the sucrose preference, novelty suppressed feeding and tail suspension tests and then, sacrificed to obtain blood samples by cardiac puncture and the brain for neuroplasticity analysis.


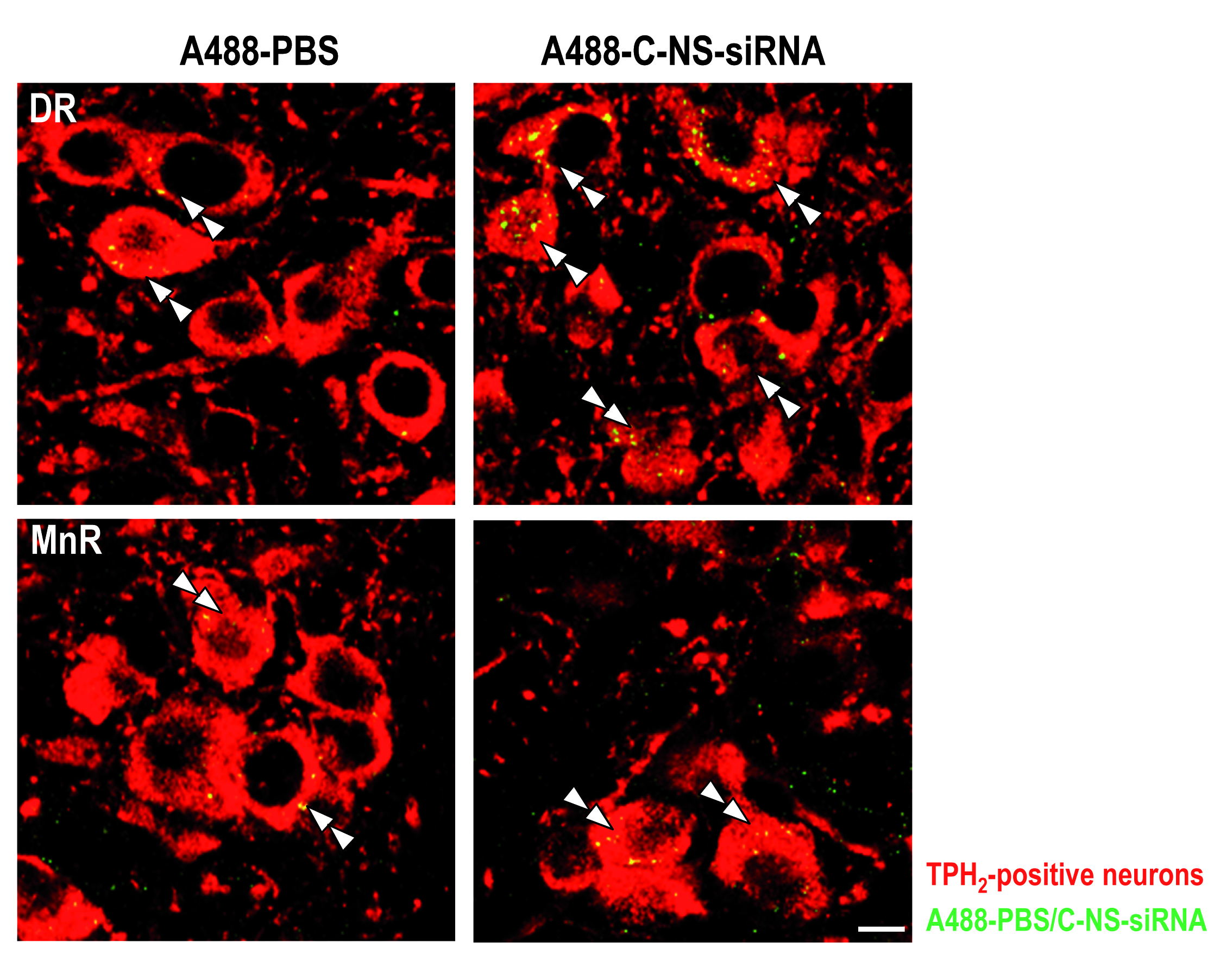


**Figure S2** Preferential accumulation of sertraline-conjugated nonsense-siRNA (C-NS-siRNA) molecules in serotonin neurons of mouse DR. Mice were intranasally administered with alexa488 PBS (A488-PBS) or alexa488-labeled C-NS-siRNA (A488-C-NS-siRNA) at 30 μg·day^-1^ during 4-day, and were killed 6h post-administration (*n*=2 mice/group). Confocal images showing co-localization of A488-PBS or A488-C-NS-siRNA (yellow) in DR and MnR 5-HT neurons (TPH_2_-positive, red) identified with double white arrowheads. Scale bars: 10 μm.

**
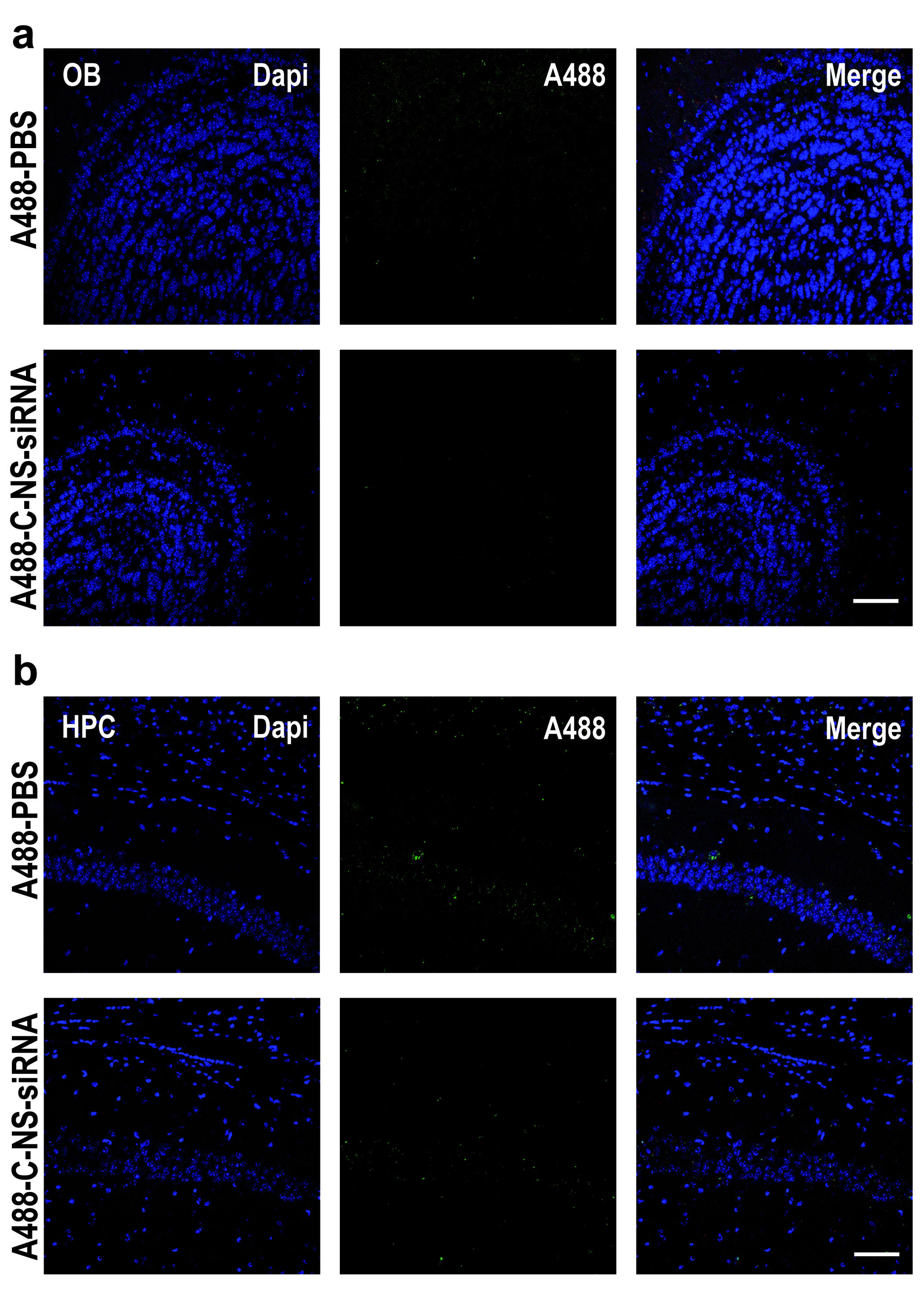
**

**Figure S3** Absence of sertraline-conjugated nonsense-siRNA (C-NS-siRNA) molecules in: **(a)** olfactory bulbs - OB and, **(b)** dorsal hippocampus - HPC of treated mice. Laser confocal images show cell nuclei stained with Dapi (blue) in OB and HPC of mice treated intranasally with vehicle or Alexa488-labeled C-NS-siRNA (A488-C-NS-siRNA) at 30 µg·day^-1^ during 4 days (*n*=3 mice/group, the same mice than in Figure 1). Scale bars: 50 µm.

**
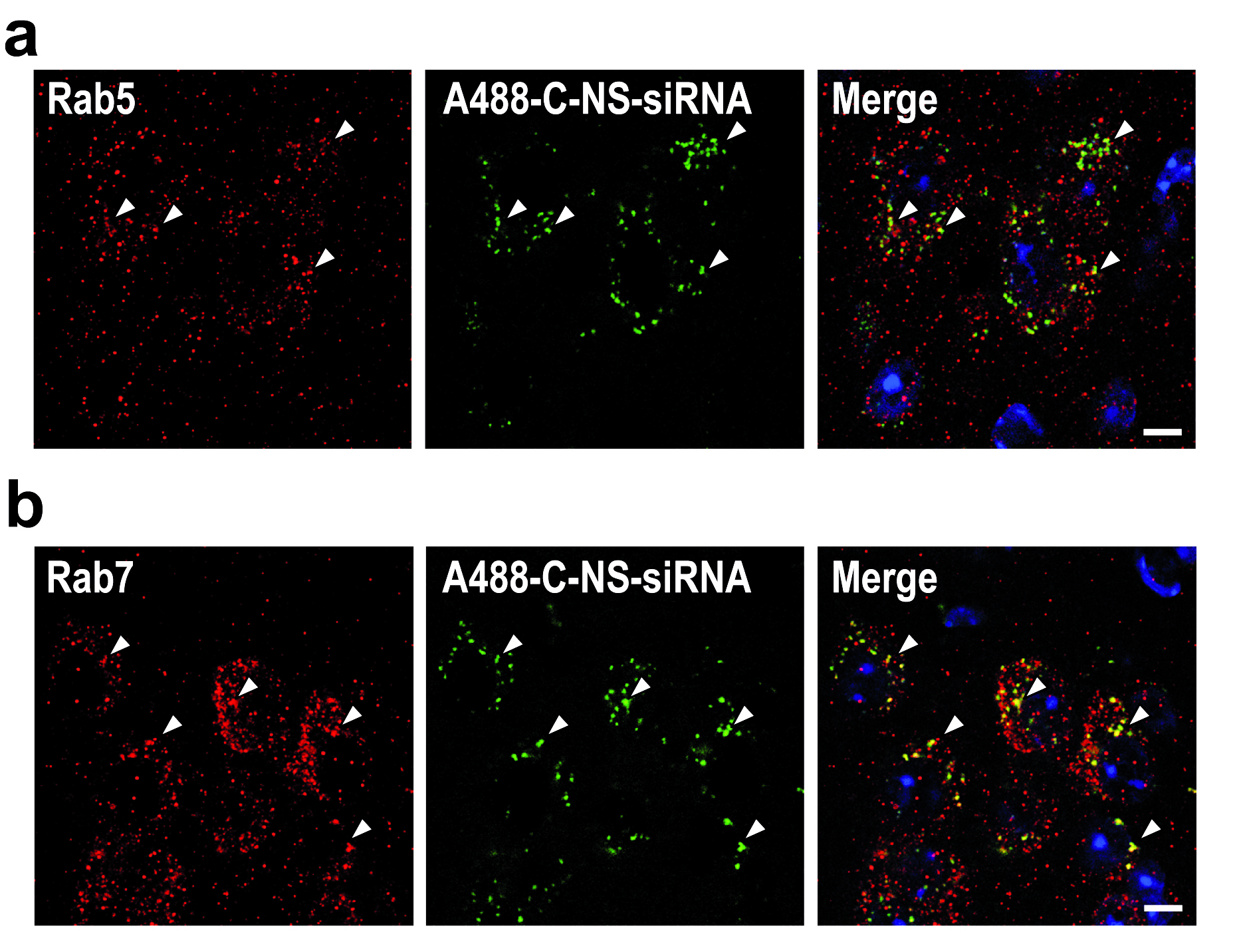
**

**Figure S4** Co-localization of alexa488-labeled sertraline-conjugated nonsense-siRNA (A488-C-NS-siRNA) with markers of endomembrane compartments. **(a)** Selected vesicles showing co-localization (yellow) between Rab5 (early endosome marker, red) with A488-C-NS-siRNA (green) in DR neurons. Scale bar: 10 μm. **(b)** Selected vesicles showing co-localization (yellow) between Rab7 (late endosome marker, red) with A488-C-NS-siRNA (green) in DR neurons. Vesicles are marked with white arrowheads. Scale bar: 10 μm.

**
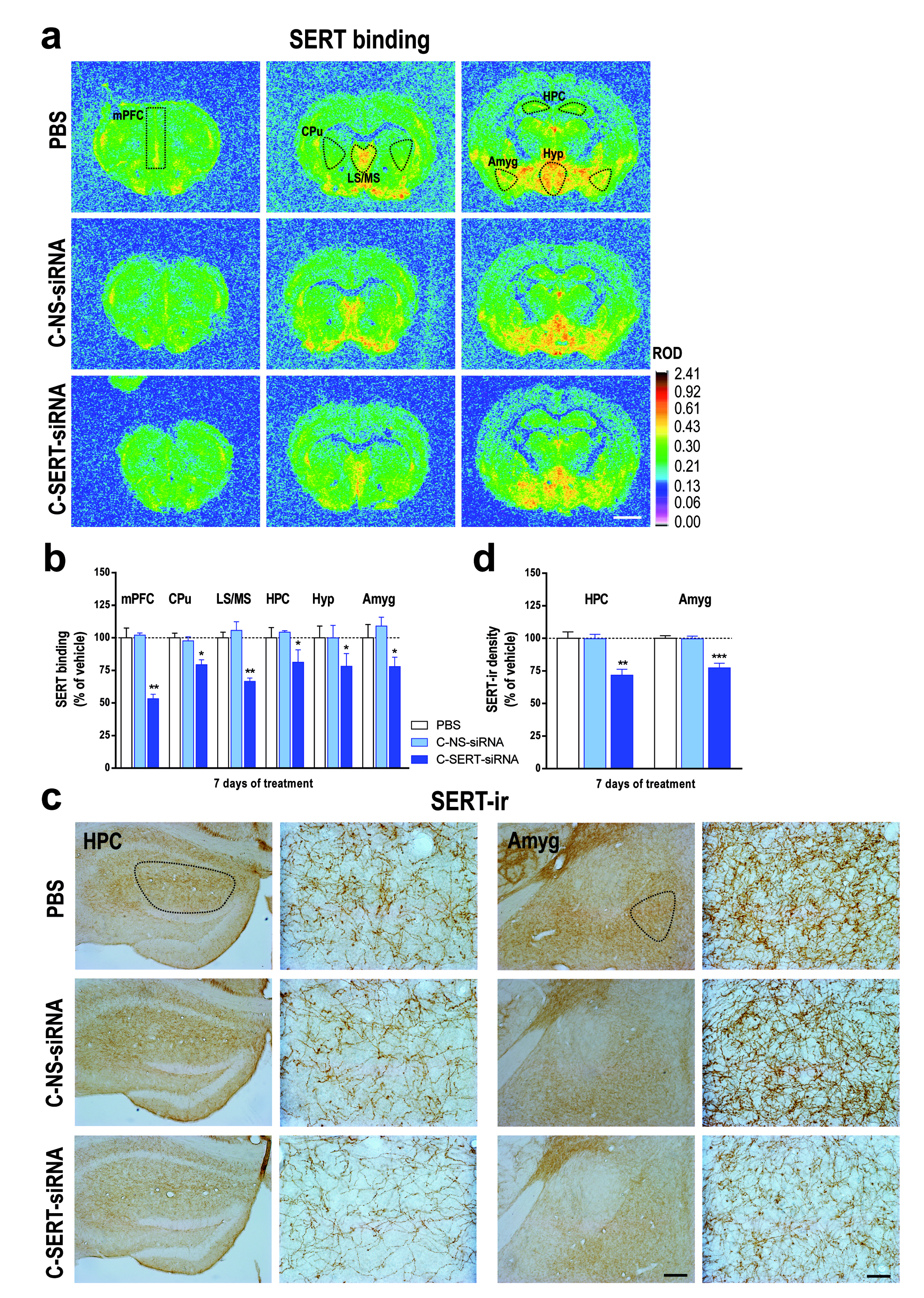
**

**Figure S5** Down-regulation of SERT binding sites and protein levels in mouse brain following sertraline-conjugated SERT-siRNA (C-SERT-siRNA). **(a)** Representative coronal brain sections showing reduced SERT binding levels in forebrain regions of mice treated with C-SERT-siRNA for 7-day. Solid line contours mark the approximate areas where densitometric analyses were performed. Medial prefrontal cortex (mPFC), caudate putamen (CPu), lateral and medial septal nuclei (LS/MS), hippocampus (HPC), hypothalamus (HYP) and, amygdala (Amyg). Scale bar: 2 mm. **(b)** Densitometric analysis of specific SERT binding is presented as % binding in the corresponding region of PBS-treated mice (*n*=4-10 mice/group; **P*<0.05, ***P*<0.01 compared to PBS and C-NS-siRNA). **(c)** Representative images of SERT-immunoreative (SERT-ir) fibers in forebrain regions such as HPC and Amyg. Images in the right hand are high-magnification photomicrographs of the frames in left hand row. Scale bars: low=100 μm, high=20 μm. **(d)** C-SERT-siRNA treatment (7-day) decreased the target SERT protein density as compared to PBS- and C-NS-siRNA-treated mice (*n*=6 mice/group; ***P*<0.01, ****P*<0.001 versus PBS and C-NS-siRNA). Bars represent mean ± s.e.m.

**
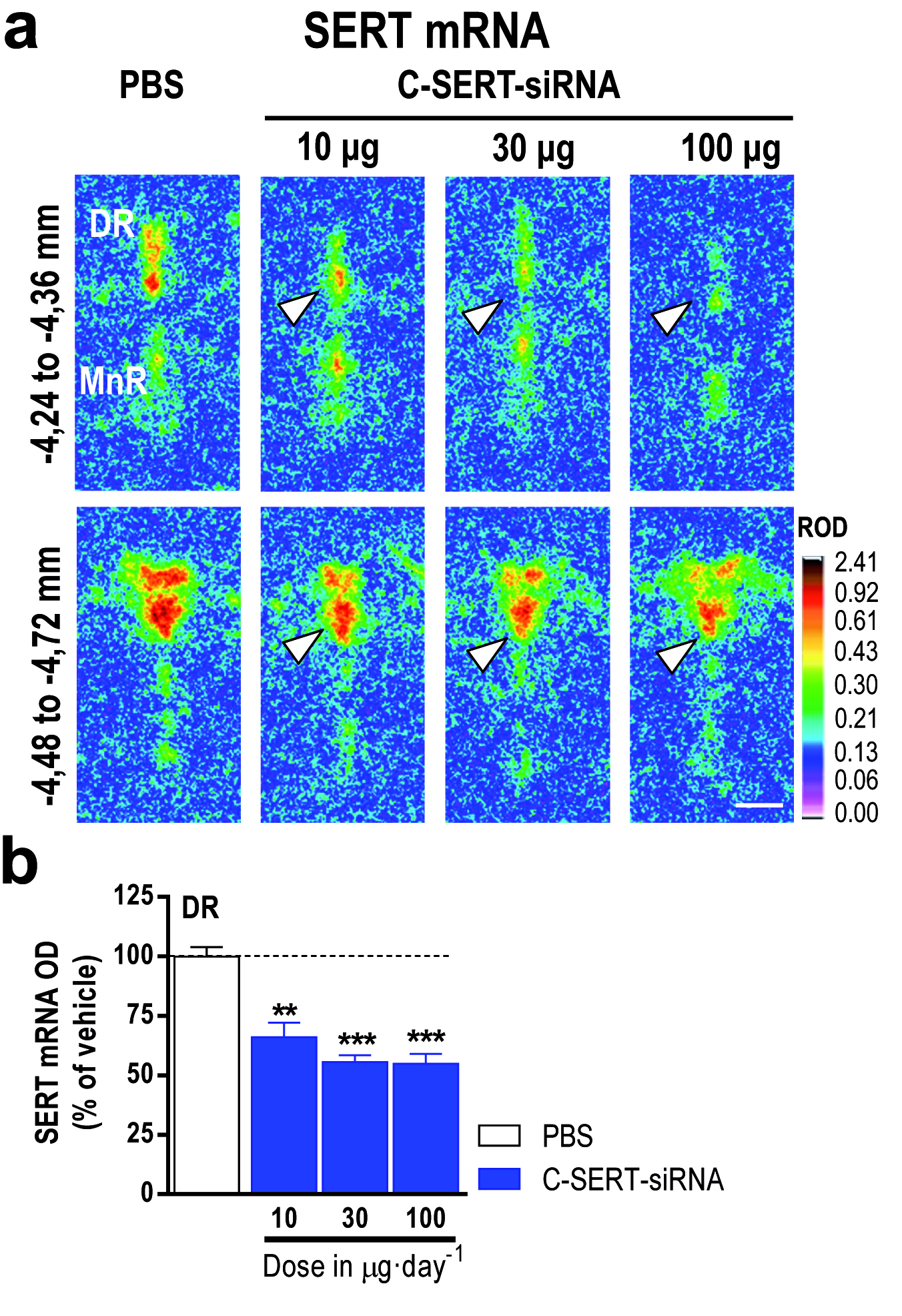
**

**Figure S6** Dose-related effects of sertraline-conjugated SERT-siRNA (C-SERT-siRNA) on SERT mRNA level in mouse DR. Mice received intranasally: PBS or C-SERT-siRNA at 10-30-100 μg·day^-1^ (0.7, 2.1 or 7 nmol·day^-1^, respectively) during 7 days and were killed 24h after last administration. **(a)** Coronal brain sections showing reduced SERT mRNA levels in the DR (AP coordinates from bregma: -4.24/-4.36 to -4.48/-4.72 in mm) of mice treated with C-SERT-siRNA (7-day) indicated with white arrowheads. Scale bar: 500 μm. **(b)** Effects of different doses of C-SERT-siRNA on SERT mRNA densities in the DR of mice (*n*=3-5 mice/group; ***P*<0.01, ****P*<0.001 versus PBS-treated mice). Bars represent mean ± s.e.m.

**
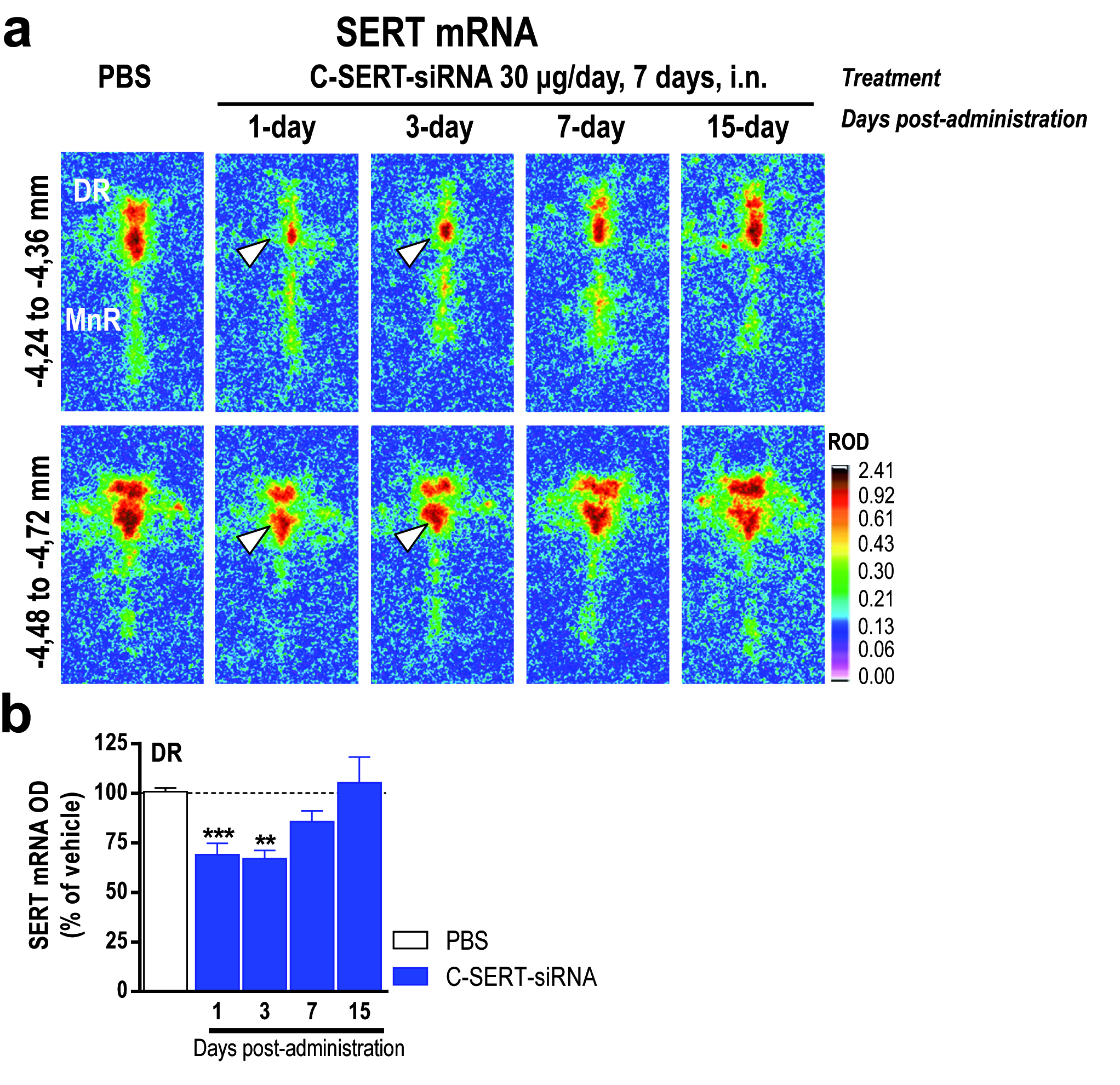
**

**Figure S7** Time course of SERT suppression in the DR after intranasal sertraline-conjugated SERT-siRNA (C-SERT-siRNA) administration. Mice received intranasally: PBS or C-SERT-siRNA at 30 μg·day^-1^ (2.1 nmol·day-1) during 7 days and were killed at 1, 3, 7 and 15 days post-administration. **(a)** Brain sections containing the raphe nuclei showing SERT mRNA density at two AP coordinates from bregma: -4.24/-4.36 to -4.48/-4.72 in mm. The arrows indicate the decreased SERT expression in the DR of mice treated with C-SERT-siRNA and sacrificed at days 1 and 3 post-treatment. Scale bar: 500 μm. **(b)** Bar graphs showing a significant reduction of SERT mRNA density in the DR of C-SERT-siRNA-treated mice compared with PBS-treated mice. Conversely, no difference was detected at days 7 and 15 post-administration (*n=*3-5 mice/group; ***P*<0.01, ****P*<0.001 versus PBS). Bars represent mean ± s.e.m.

**
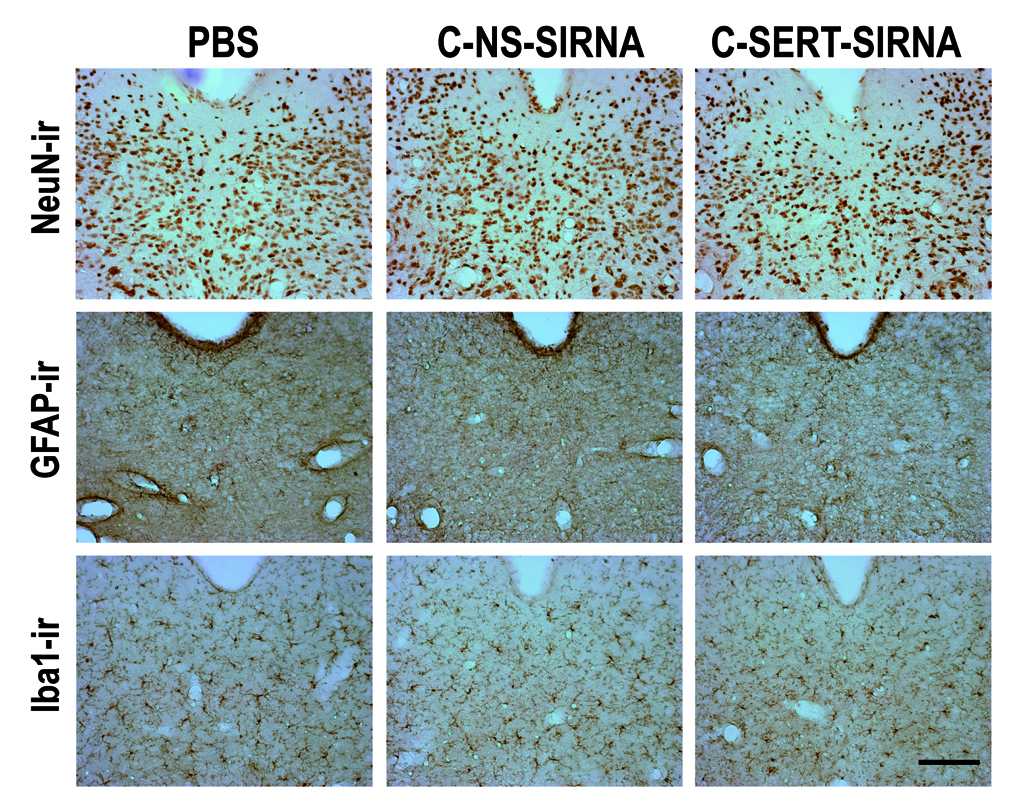
**

**Figure S8** Immunohistochemical assessment of cellular viability in the raphe nuclei after intranasal administration of sertraline-conjugated SERT-siRNA (C-SERT-siRNA). Mice received intranasally PBS, C-NS-siRNA or C-SERT-siRNA at 30 μg·day^-1^ during 7 days (*n*=4 mice/group). Adjacent 30-μm-thick sections through the midbrain raphe nuclei were stained with neuronal NeuN, astrocytic GFAP or, microglial Iba1 markers. No differences were found between all experimental groups for any of the markers. Scale bar: 100 μm.

**
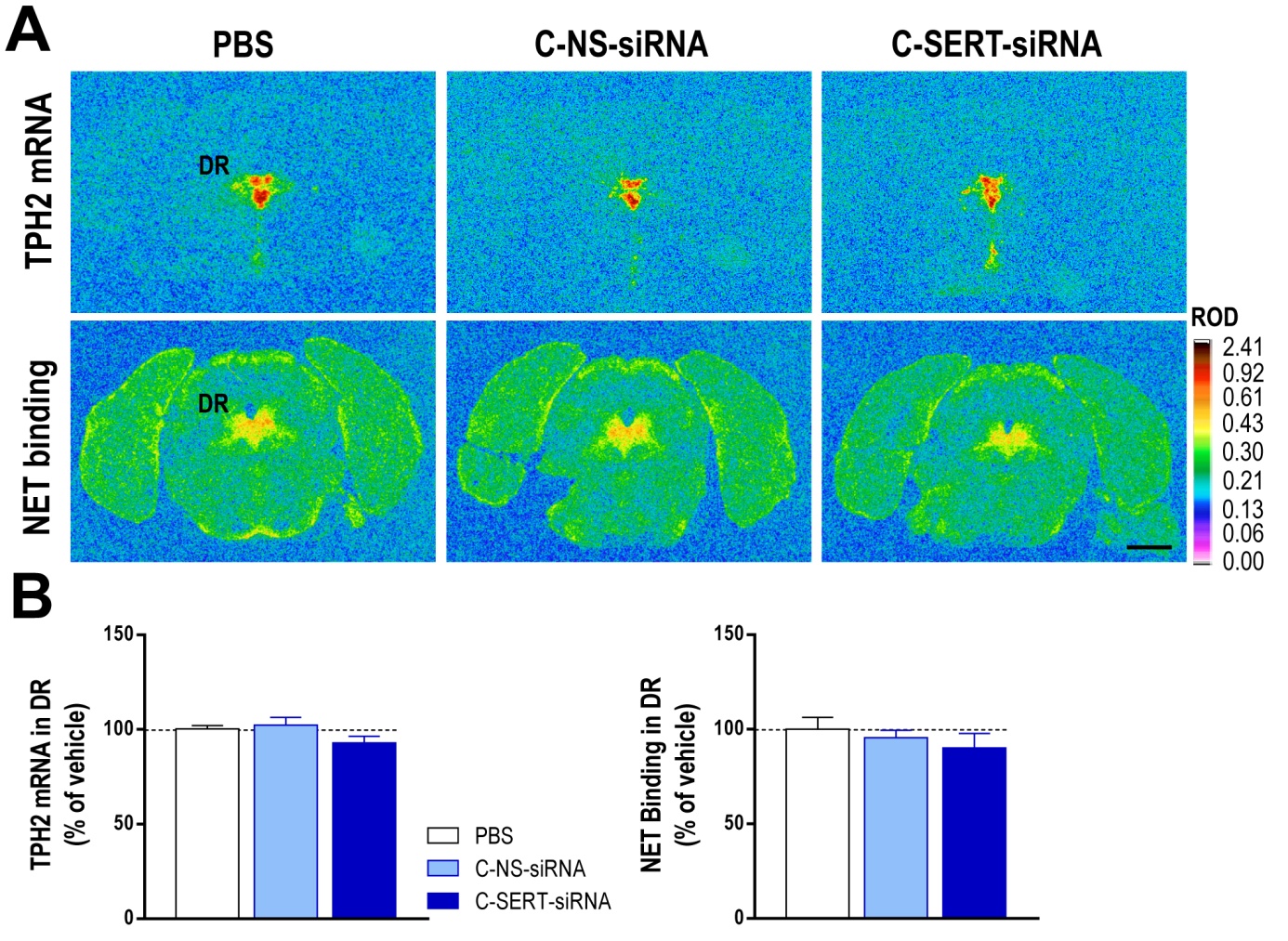

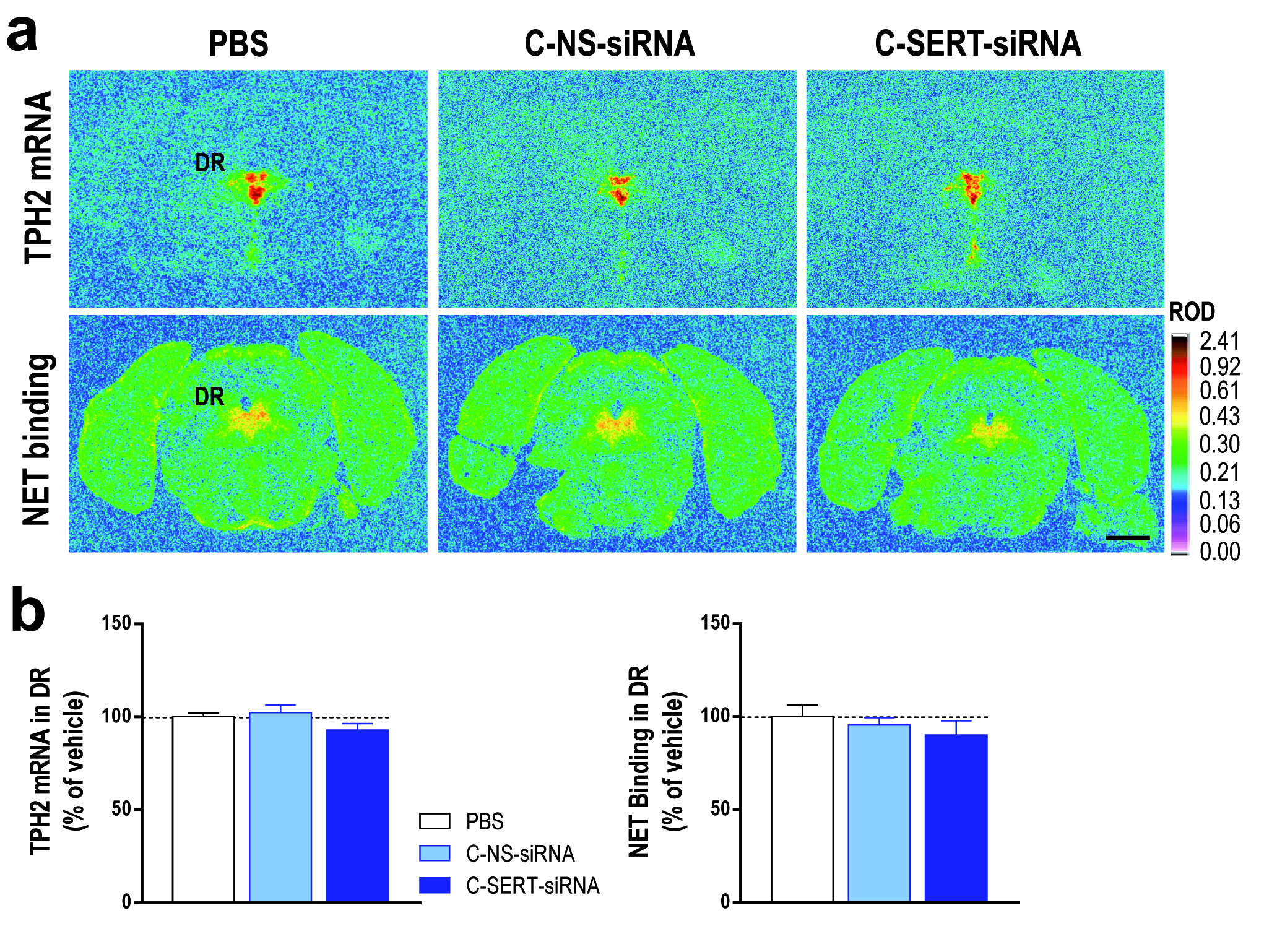
**

**Figure S9** Selectively of SERT expression silencing after intranasal administration of sertraline-conjugated SERT-siRNA (C-SERT-siRNA). Mice received intranasally PBS, sertraline-conjugated nonsense-siRNA (C-NS-siRNA) or C-SERT-siRNA at 30 μg·day^-1^ during 7-day. **(a)** Representative coronal midbrain sections showing tryptophan hydroxylase-2 (TPH2, rate-limiting enzyme for 5-HT synthesis) mRNA density and [^3^H]-nisoxetine binding to the norepinephrine transporter (NET) in the dorsal raphe nuclei (DR). Scale bar: 2 mm. **(b)** Bar graphs showing no differences in TPH2 mRNA levels and NET binding sites in the DR (*n*=3-4 mice/group). Values are mean ± s.e.m.

**
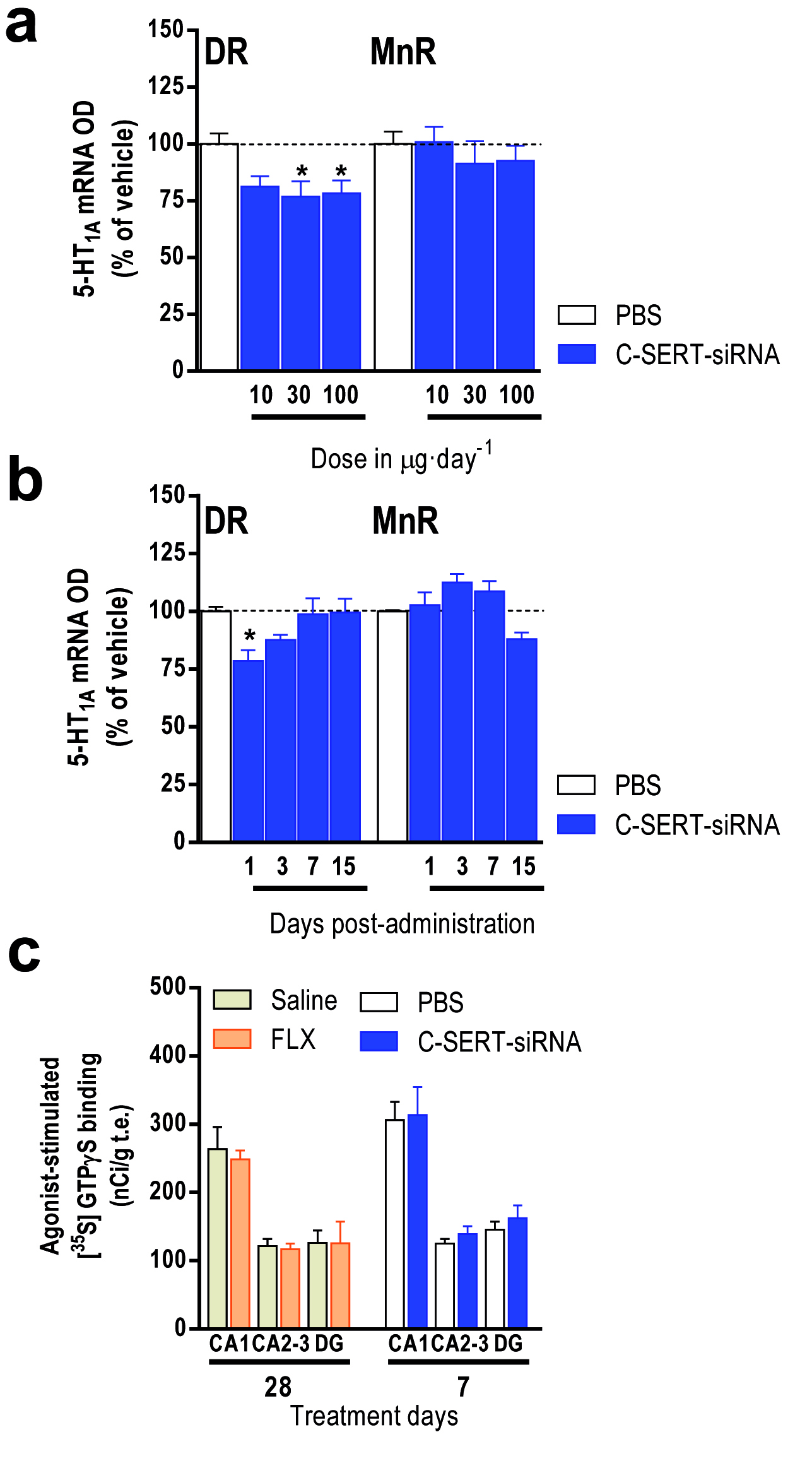
**

**Figure S10 (a)** Bar graphics showing effects of different doses of sertraline-conjugated SERT-siRNA (C-SERT-siRNA) on 5-HT_1A_ mRNA densities in the mouse DR and MnR (*n*=3-5 mice/group; **P*<0.05 versus PBS-treated mice). Mice received intranasally PBS or C-SERT-siRNA at 10-30-100 μg·day^-1^ during 7-day and were killed a 24h after last administration. **(b)** Bar graphs showing a significant reduction of 5-HT_1A_ mRNA density in the DR of C-SERT-siRNA-treated mice (30 μg·day^-1^, 7-day) versus PBS-treated mice at 1 day post-administration. Conversely, no difference was detected at days 3, 7 and 15 post-administration (*n*=3-12 mice/group; **P*<0.05 versus PBS). **(c)** Effects of C-SERT-siRNA (30 μg·day^-1^, 7-day) and FLX (10 mg·Kg^-1^, 28-day) on post-synaptic 5-HT_1A_ receptor function in the hippocampus (*n*=5-10 mice/group). Values are mean ± s.e.m.

**
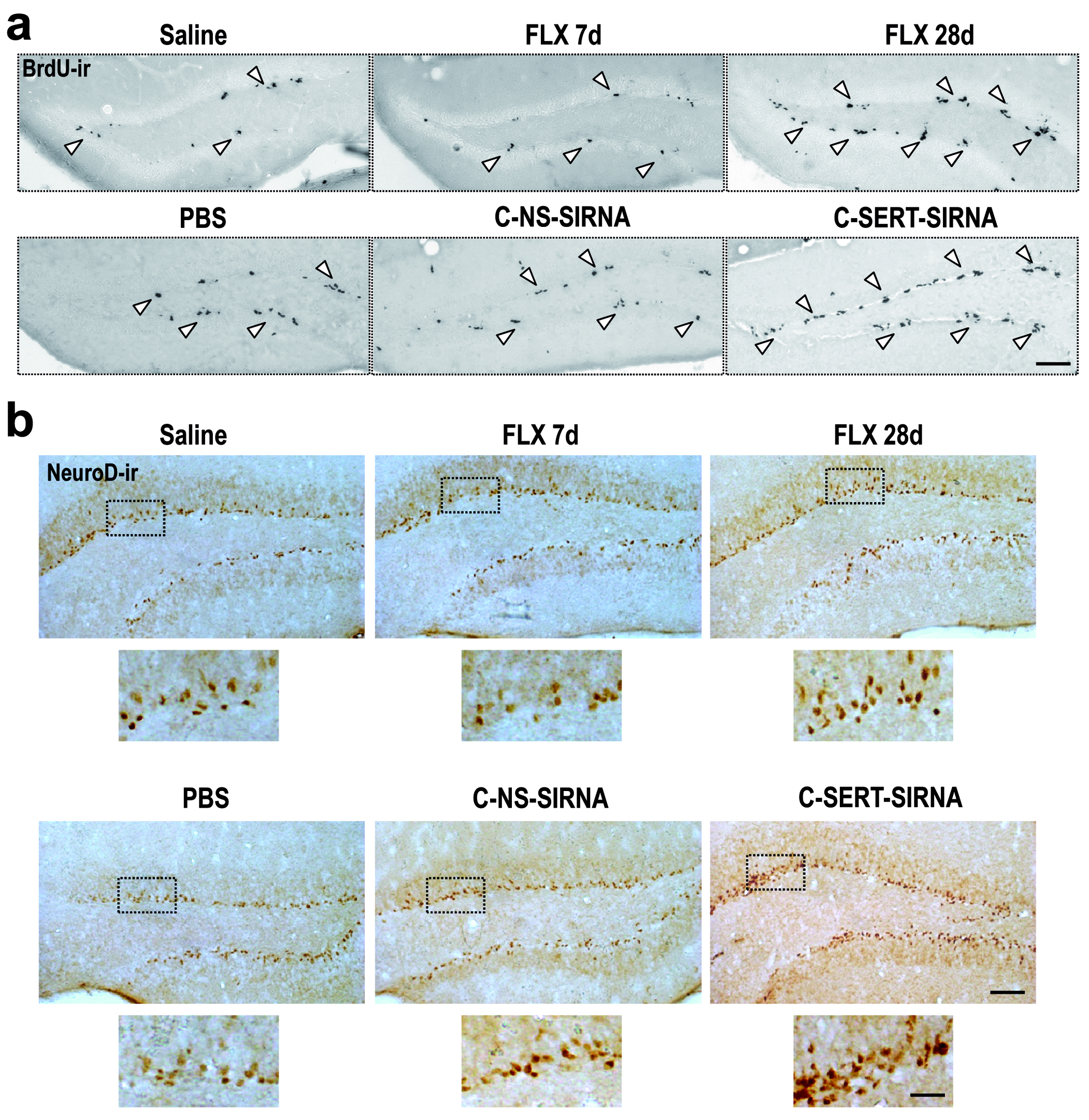
**

**Figure S11** Short-term sertraline-conjugated SERT-siRNA (C-SERT-siRNA) stimulates the proliferation of cell precursors and immature neurons. Mice were intranasally administered with: PBS, sertraline-conjugated nonsense-siRNA (C-NS-siRNA) or C-SERT-siRNA at 30 μg·day during 7-day. Other groups of mice were treated with saline or fluoxetine (FLX) at 10 mg·kg·day, i.p. during 7- or 28-day. **(a)** Representative images showing an increased number of BrdU-positive cells in the dentate gyrus (DG) of C-SERT-siRNA (7-day) or FLX-treated mice (28-day), but not FLX 7-day, compared to their respective control mice. White arrowheads mark some BrdU-positive cells. Scale bar: 100 μm. **(b)** Immunohistochemical images showing NeuroD-positive progenitors in the DG of mice. Bottom row are high-magnification photomicrographs of the frames in top row. Scale bars: low=100 μm and high=20 μm.

**
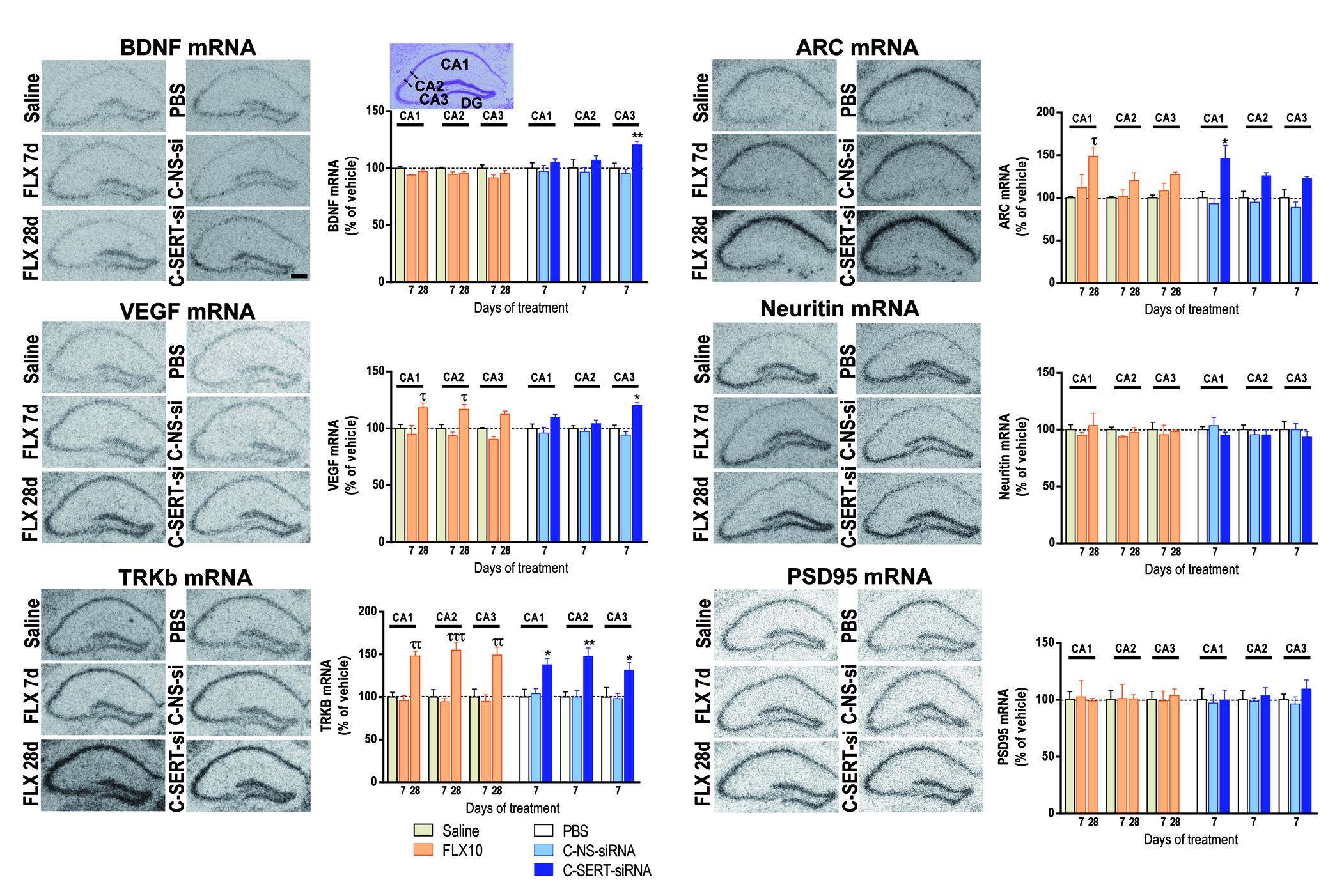
**

**Figure S12** Expression of neuroplasticity genes in the different mouse hippocampal subfields including CA1, CA2 and CA3. Mice were intranasally administered with: PBS, sertraline-conjugated nonsense-siRNA (C-NS-siRNA) or C-SERT-siRNA at 30 μg·day^-1^ during 7-day. Other groups of mice were treated with saline or fluoxetine (FLX) at 10 mg·kg^-1^·day^-1^, i.p. during 7- or 28-day. Representative autoradiograms of hippocampal sections of mice are shown BDNF, VEGF, TRKB, ARC, Neuritin and PSD95 mRNA expression. Scale bar: 100 μm. Densitometric analyses were performed in different hippocampal regions: CA1, CA2 and CA3 shown in the cresyl violet-stained section (top). Levels of mRNA for each gene are shown in the bar graphs next to the representative autoradiograms (*n*=3-8 mice/group; **P*<0.05, ***P*<0.01 versus PBS and C-NS-siRNA; ^𝞃^*P*<0.05, ^𝞃𝞃^*P*<0.01, ^𝞃𝞃𝞃^*P*<0.001 versus saline FLX 7-day). Values are mean ± s.e.m.

**
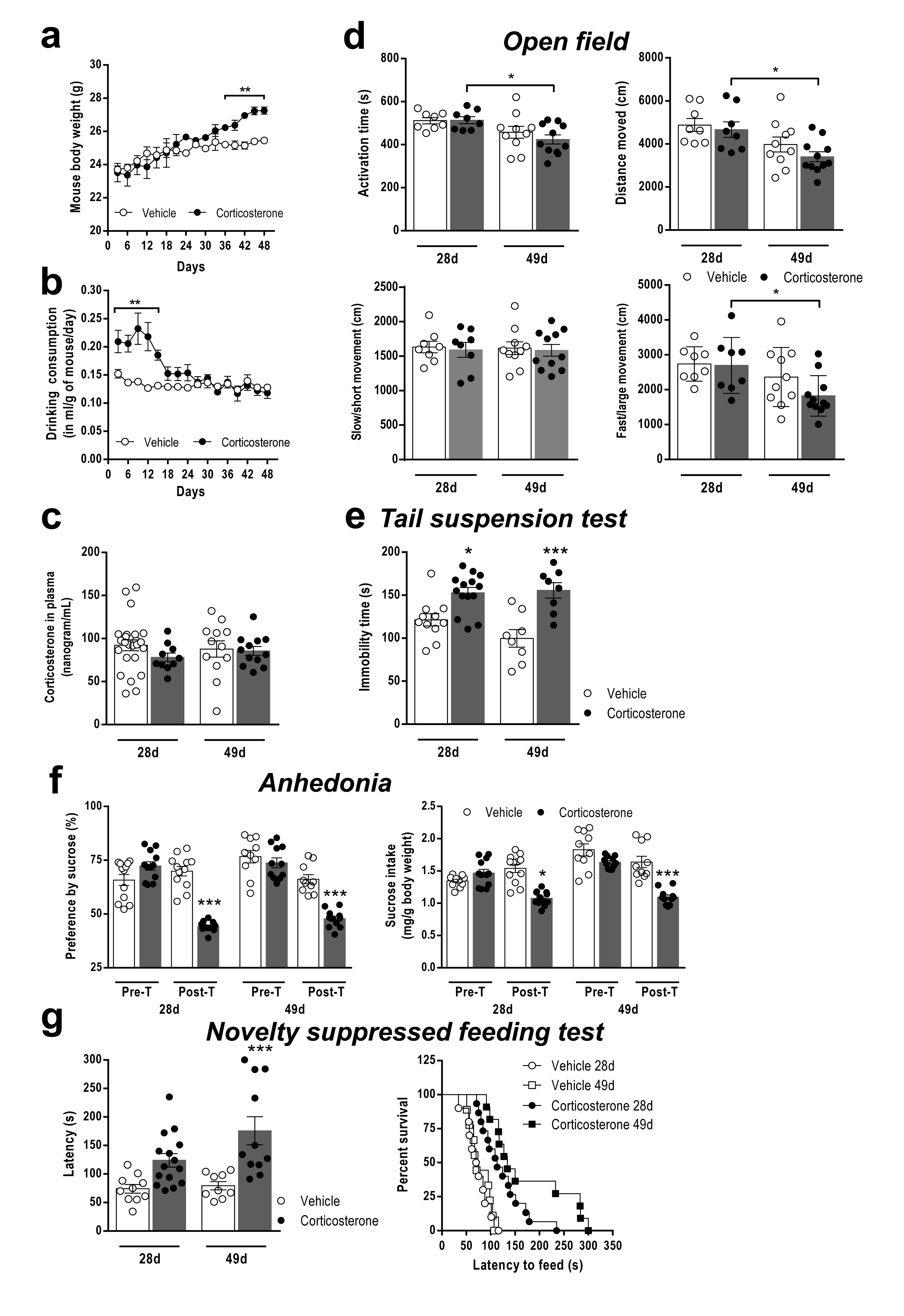
**

**
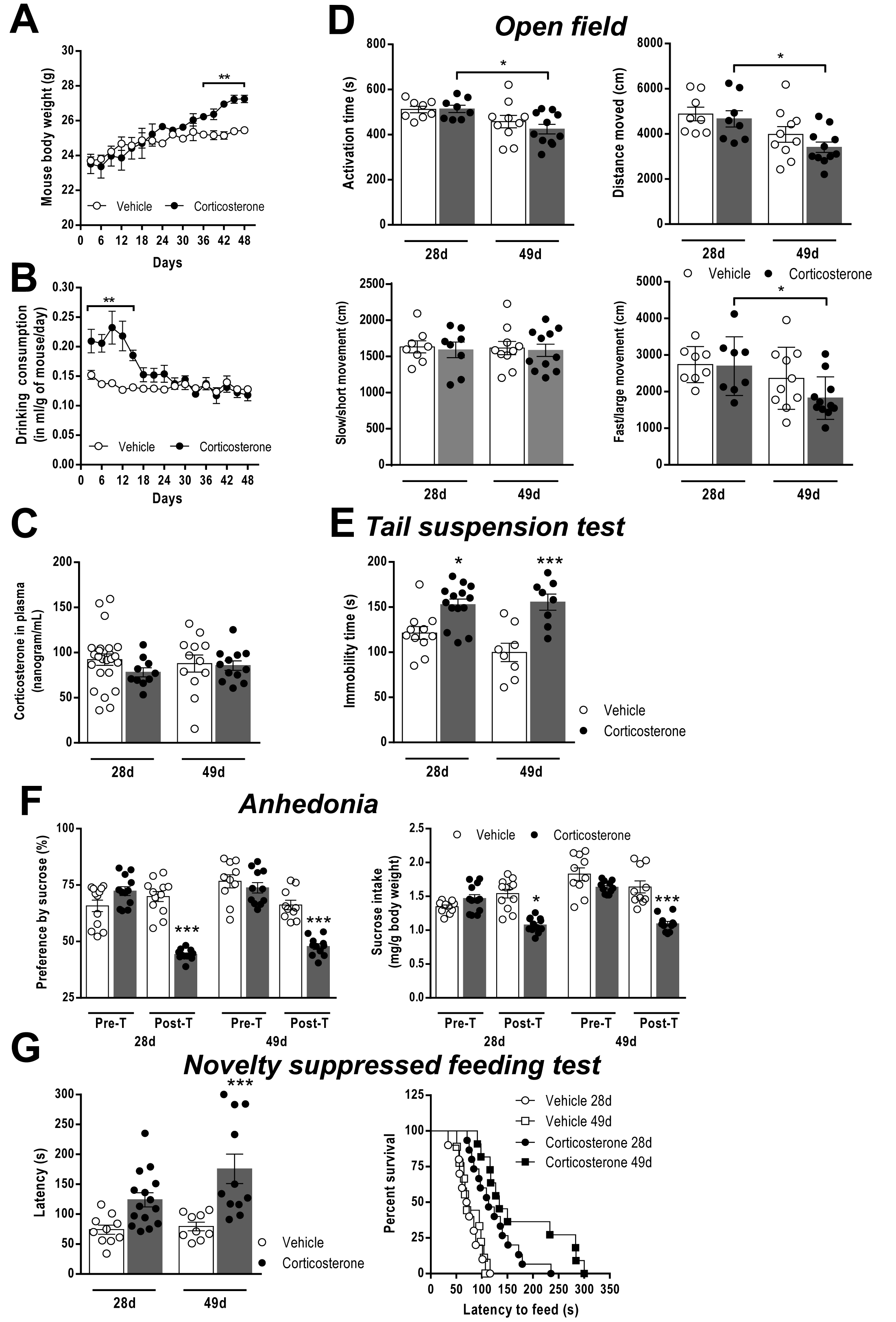
**

**Figure S13** Effects of chronic corticosterone exposure on body weight, drinking of the corticosterone solution, plasma corticosterone levels and behavioral assessments. **(a)** Corticosterone-exposed mice showed an increase of body weight from day 36 onwards (*n*=2-6 groups of 5 mice/each; ***P*<0.01 versus vehicle-exposed mice). **(b)** Fifteen-day corticosterone treatment (30 μg/ml) increased the consumption of the corticosterone solution, yet drinking returned to control values after reducing corticosterone dosage (15 and 7.5 μg·ml^-1^) (*n*=2-16 groups of 5 mice/each; ***P*<0.01 versus vehicle-exposed mice). **(c)** Plasma corticosterone levels did not differ between groups (*n*=12-24 mice/group). **(d)** Effects of the two corticosterone regimes (28- or 49-day) on locomotor activity in the open field test. No differences were observed in the activation time, distance moved, slow/short and fast/large movement between vehicle and corticosterone-exposed mice (*n*=8-11 mice/group). However, a significant effect of corticosterone regimen was detected in the corticosterone group for activation time, distance moved and fast/large movements (**P*<0.05). **(e)** Effects of the two corticosterone regimes (28- or 49-day) on tail suspension test (*n*=8-15 mice/group; **P*<0.05, ****P*<0.001 versus vehicle-exposed mice). **(f)** Effects of the two corticosterone regimes (28- or 49-day) on anhedonia. Results are expressed as mean of % preference by sucrose or mean of sucrose intake (*n*=10-12 mice/group; ****P*<0.001 versus vehicle-exposed mice). No difference in sucrose preference before corticosterone pre-treatment (at baseline measurement) was observed. **(g)** Effects of the two corticosterone regimes (28- or 49-day) on the novelty suppressed feeding test. Results are expressed as mean of latency to feed (in seconds) or cumulative survival (*n*=9-15 mice/group; ****P*<0.001 versus vehicle-exposed mice). No differences were detected in the feeding drive of each mouse after returning the animal to the familiar environment in the home cage, immediately after the test. Values are mean ± s.e.m.

**Supplementary Tables**

**Table S1.** Sequences of siRNA molecules used

| **siRNA** | **forward** | **Reverse** |
| --- | --- | --- |
| **SERT-siRNA** | GCUAGCUACAACAAGUUCATT | UGAACUUGUUGUAGCUAGCTT |
| **NS-siRNA** | AGUACUGCUUACGAUACGGTT | CCGUAUCGUAAGCAGUACUTT |

**Table S2.** Summary of the conditions for labeling 5-HT_1A_R, SERT and NET in the present work

| **Protein** | **Ligand** | **[nM]** | **Buffer** | **Pre-Inc**  **(RT, min)** | **Inc**  **Buffer** | **Inc**  **(RT, min)** | **Washing**  **(min)** | **Exposure**  **(days)** | **Blank** |
| --- | --- | --- | --- | --- | --- | --- | --- | --- | --- |
| **5-HT_1A_** | [^3^H]-8-OH-DPAT | 1.0 | A | 30 | A +10μM  pargyline | 60 | 2 x 5 | 60 | 10μM  serotonin |
| **SERT** | [^3^H]-citalopram | 1.5 | B | 15 | B | 60 | 2 x 10 | 45 | 1μM  fluoxetine |
| **NET** | [^3^H]-nisoxetine | 3.0 | C | 15 | C | 240 | 3 x 5 | 90 | 10μM  mazindol |

RT, room temperature; IC, ice-cold buffer; **Buffer A**: 170mM Tris-HCl, 4mM CaCl_2_, 0.01% ascorbic acid, pH 7.6; **Buffer B**: 50mM Tris-HCl, 120mM NaCl, 5mM KCl, pH 7.6; **Buffer C**: 50mM Tris-HCl, 300mM NaCl, 5mM KCl, pH 7.4.

**Table S3.** Summary of complete statistical analyses

Excel file

**Table S4.** Number of immunoreactive-positive cells in the different treatments

| **Treatments** | **Total Ki67-**  **positive cells** | **Total BrdU-**  **positive cells** | **NeuroD-**  **positive cells/mm^2^** | **DCX-**  **positive cells/mm^2^** |
| --- | --- | --- | --- | --- |
| **Saline 7d** | 2196 ± 283 | 3882 ± 385 | 122 ± 12 | 99 ± 3 |
| **FLX 7d** | 2264 ± 122 | 3599 ± 162 | 130 ± 15 | 100 ± 5 |
| **Saline 28d** | 1195 ± 83 | 3242 ± 78 | 91 ± 9 | 85 ± 2 |
| **FLX 28d** | 1719 ± 138* | 4368 ± 433* | 131 ± 12* | 100 ± 4* |
| **PBS 7d** | 2639 ± 229 | 3826 ± 106 | 134 ± 15 | 145 ± 6 |
| **C-NS-siRNA 7d** | 2077 ± 333 | 3755 ± 200 | 128 ± 17 | 141± 4 |
| **C-SERT-siRNA 7d** | 3830 ± 347* ^𝞃 𝞃^ | 5481 ± 292** ^𝞃 𝞃^ | 194 ± 12* ^𝞃^ | 171 ± 7* ^𝞃 𝞃^ |

Mice were intranasally administered with: PBS, sertraline-conjugated nonsense-siRNA (C-NS-siRNA) or sertraline-conjugated SERT-siRNA (C-SERT-siRNA) at 30 μg·day^-1^ during 7-day. Other groups of mice were treated with saline or fluoxetine (FLX) at 10 mg·kg^-1^·day^-1^, i.p. during 7- or 28-day (*n*=5-11 mice/group; **P*<0.05, ***P*<0.01 versus saline or PBS, respectively; ^𝞃^*P*<0.05, ^𝞃𝞃^*P*<0.01 versus C-NS-siRNA). Values are mean ± s.e.m.
